# The RNA landscape of *Dunaliella salina* in response to short-term salt stress

**DOI:** 10.3389/fpls.2023.1278954

**Published:** 2023-12-04

**Authors:** Bingbing Zhang, Caiyun Deng, Shuo Wang, Qianyi Deng, Yongfan Chu, Ziwei Bai, Axiu Huang, Qinglian Zhang, Qinghua He

**Affiliations:** ^1^ The Research Institute of Qinghai-Tibet Plateau, Southwest Minzu University, Chengdu, China; ^2^ School of Laboratory Medicine, Chengdu Medical College, Chengdu, China; ^3^ Key Laboratory of Qinghai-Tibet Plateau Animal Genetic Resource Reservation and Utilization, Southwest Minzu University, Chengdu, China

**Keywords:** salt stress, *Dunaliella salina*, comparative transcriptomic analysis, protein Folding, DNA repair, cellular redox homeostasis

## Abstract

Using the halotolerant green microalgae *Dunaliella salina* as a model organism has special merits, such as a wide range of salt tolerance, unicellular organism, and simple life cycle and growth conditions. These unique characteristics make it suitable for salt stress study. In order to provide an overview of the response of *Dunaliella salina* to salt stress and hopefully to reveal evolutionarily conserved mechanisms of photosynthetic organisms in response to salt stress, the transcriptomes and the genome of the algae were sequenced by the second and the third-generation sequencing technologies, then the transcriptomes under salt stress were compared to the transcriptomes under non-salt stress with the newly sequenced genome as the reference genome. The major cellular biological processes that being regulated in response to salt stress, include transcription, protein synthesis, protein degradation, protein folding, protein modification, protein transport, cellular component organization, cell redox homeostasis, DNA repair, glycerol synthesis, energy metabolism, lipid metabolism, and ion homeostasis. This study gives a comprehensive overview of how *Dunaliella salina* responses to salt stress at transcriptomic level, especially characterized by the nearly ubiquitous up-regulation of the genes involving in protein folding, DNA repair, and cell redox homeostasis, which may confer the algae important mechanisms to survive under salt stress. The three fundamental biological processes, which face huge challenges under salt stress, are ignored by most scientists and are worth further deep study to provide useful information for breeding economic important plants competent in tolerating salt stress, other than only depending on the commonly acknowledged osmotic balance and ion homeostasis.

## 1 Introduction

Salt stress of plants is an active area of research. A lots of researches have been carried out using the model plant *Arabidopsis thaliana* (Møller and Tester, 2007), partially because that *Arabidopsis thaliana* has a clear genetic background and is from the same family *Brassicaceae* as some economic important crops and vegetables, such as rapeseed, cabbage, radish et al., and the response mechanisms of *Arabidopsis thaliana* under salt stress may better reflect those of related crops (Kowalski et al., 1994).

*Dunaliella salina* is an extremely halotolerant, unicellular, eukaryotic, photosynthetic green microalgae, which is unique in its remarkable ability to survive in media containing NaCl at a wide range of concentrations, from about 0.05 M to 5.5 M (Ben-Amotz and Avron, 1973). *Dunaliella salina* can grow easily in aqueous media in a flask, which makes it very convenient to apply salt stress and other abiotic stresses, such as heavy metal, nutrition, temperature and light stress, on it for study. Compared with *Arabidopsis thaliana*, *Dunaliella salina* is unicellular, cells in log phase are highly homogeneous. For this reason, it is hopefully to find some of the fundamental and conserved mechanisms that remain uncovered due to cellular heterogeneity of study materials. These characteristics make *Dunaliella salina* a very good model organism for studying salt stress response and tolerance (Ben-Amotz and Avron, 1973).

Omics methods, such as methods of genomics, transcriptomics and proteomics, are powerful tools to reveal the mechanisms of salt tolerance and can give an overview of the response of plants to salt stress because omics aims at the collective characterization and quantification of pools of biological molecules (Mangelsen et al., 2011; Marco et al., 2011; Kang et al., 2020). With the development of high throughput sequencing technology, comparative transcriptomic analysis becomes an efficient and powerful method to reveal the response of plants to all kinds of stress at a global level, and some papers reported using this method to study the response of *Dunaliella salina* to salt stress (He et al., 2020a; Gao et al., 2021; Lv et al., 2021; Panahi and Hejazi, 2021). Considering that morphological change and glycerol synthesis in *Dunaliella salina* are almost accomplished in about 2 hours after salt stress (Chitlaru and Pick, 1989; Oren, 2005), it is universally acknowledged that most of the adaptive changes of *Dunaliella salina* should occur within about 2 hours under salt stress. Lv et al. (Lv et al., 2021) reported the transcriptomes at 15, 30, 60, and 120-min under salt stress of 4.5 M NaCl, finding that GO (Gene Ontology) terms of “chromosome and associated proteins”, “transporters”, and “cytoskeleton proteins” were enriched by differentially expressed genes (DEGs). They also analyzed the expression of enzymes in the core carbon metabolism pathway, such as starch catabolism, glycolysis, Calvin cycle and glycerol metabolic pathways. However, except the core carbon metabolism pathway, other biological processes and pathways weren’t analyzed in detail. He et al. (He et al., 2020a) reported the transcriptomes at 30, 60, and 120-min under salt stress of 2.5M NaCl, found that some biological processes, such as photosynthesis, lipid metabolism, amino acids and protein metabolism, starch and sucrose metabolism and glycerol synthesis, were enriched by the DEGs. They also constructed for the first time the carbon metabolism pathway from starch to glycerol in *Dunaliella salina*. In that study, the transcripts from the third-generation sequencing (PacBio sequencing) was used as the reference genes for gene expression quantification analysis due to there was no genome data of *Dunaliella salina* strain CCAP 19/30 available at the moment. Although this method has the advantage to detect alternatively spliced transcripts, there exist obvious drawbacks, since artificially spliced transcripts generated during incomplete reverse transcription could interfere the expressional quantification of the genes and the potential loss of low-abundance mRNAs could lower genome coverage (Van Dijk et al., 2018). Recently we sequenced the whole genome of *Dunaliella salina* strain CCAP 19/30 by PacBio sequencing, which gave a total of 13509 genes by annotation. Using this high-quality genome sequence as the reference, we redo in this study the comparative transcriptomic analysis for *Dunaliella salina* strain CCAP 19/30 under salt stress of 2.5 M NaCl within 2 hours duration. In addition to helping to focus on identifying and analyzing the major biological processes being regulated to reflect the responses of *Dunaliella salina* to salt stress at a global level, the high-quality reference genome also enables us to analyze the specific pathways, the glycerol synthesis, the fatty acid synthesis, and the TAG synthesis pathway in detail, especially the key enzymes that regulate a specific pathway. This work gives an overview of the response of *Dunaliella salina* to salt stress at transcriptomic level. The nearly ubiquitous up-regulation of the genes involved in protein folding, DNA repair, and cell redox homeostasis characterizes the expression profile of *Dunaliella salina* when confronting salt stress, which may be important mechanisms to enable the algae to survive under salt stress.

## 2 Materials and methods

### 2.1 Algae and culture conditions

*Dunaliella salina* strain CCAP 19/30 was obtained from Mariela A. González and Thomas Pröschold. The algae grew in a controlled-environment chamber with 16 h lighting (17500 lx) and 8 h darkness at 20 °C. The growth medium contains 1 M NaCl, 5.0 mM NaNO_3_, 5.0 mM MgSO_4_ · 7H_2_O, 0.1 mM NaH_2_PO_4_ · 2H_2_O, 1.0 mM KCl, 10.0 mM NaHCO_3_, 0.3 mM CaCl_2_ · 2H_2_O, 4.6 uM H_3_BO_3_, 0.9 uM MnCl_2_.4H_2_O, 0.08 uM ZnSO_4_.7H_2_O, 0.03 uM CuSO_4_.5H_2_O, and 0.02 uM Na_2_MoO_4_.2H_2_O. Briefly, the algae grew in growth medium containing 1 M NaCl. Salt stress was applied by adding equal volume of high-salt medium (containing 4M NaCl) to the growth medium containing algae in log phase (1 × 10^6^ cells/ml), which resulted in an increasing of salt concentration from 1 M to 2.5 M. Then the algae were collected at the time points of 0.5 h, 1 h and 2 h after the applying of salt stress, respectively, for RNA extraction. The algae were also collected before stress for RNA extraction to serve as the control. The salt stress and RNA extraction experiments were performed in triplicate.

### 2.2 Genome sequencing, assembling, and annotation

The genomic DNA was extracted using plant genomic DNA extraction kit (DP305, TIANGEN, China) by omitting the step of tissue homogenization by liquid nitrogen. Following genomic DNA extraction, libraries were generated and sequenced by using either the Illumina (insert size 350 bp) or the PacBio platforms. Totally, 102.65 Gbp were produced including 25.44 Gbp Illumina data (sequencing depth is 92.7 × and 77.21 Gbp PacBio data (sequencing depth is 281.4 × The sequencing coverage is 99.38%. The genome was assembled using overlap-layout-consensus algorithm (Lieberman-Aiden et al., 2009). The main genome assembly is about 223.7 Mbp with contig N50 = 2.93 Mbp, scaffold N50 = 13.13 Mbp. Using the RNA-Seq data of *Dunaliella salina* strain CCAP 19/30 and the published genomic data of closely related algae, 13509 protein-coding genes were found in the genome and used for comparative transcriptomic analysis.

### 2.3 RNA-Seq and gene expression analysis

Total RNA was extracted with Trizol (Invitrogen, USA) by following the user manual for suspension cell culture. Briefly, the algae cells from salt stress treatment group and control group were collected immediately by centrifugation at room temperature for 5 min at 4000×g , and quickly lysed by adding 1 ml Trizol. The time from centrifugation to lysing should be as short as possible to avoid additional stress. The remaining steps are the same as the manual recommends. The RNA pellets were kept in 75% ethanol and stored at -80°C before library construction. For library construction, mRNA was purified from total RNA and fragmented to perform cDNA synthesis. The cDNA was used to construct library with insert size of 350 bp. The libraries were sequenced by HiSeq 2000 platform to generate paired-end reads of 150 bp. The raw reads were processed to generate clean reads by removing adapter sequences, excluding reads contain >10% ambiguous bases N and > 50% bases with Qphred ≤20. The clean reads were used to quantify the values of expression of the reference genes from genome annotation of *Dunaliella salina* CCAP 19/30. Briefly, read count of each gene was calculated by mapping the clean reads from each RNA sample to the full-length coding sequence of the reference gene using HTSeq v0.6.1 (Anders et al., 2015). And then FPKM (expected number of Fragments per Kilobase of transcript sequence per Millions base pairs sequenced) of the genes was calculated by involving parameters of gene length, reads count and sequencing depth (Trapnell et al., 2010).

Differential expression analysis of the genes from two groups was performed by using the DESeq R package with the read counts of the genes (Anders and Huber, 2010). The resulting P values from DESeq were adjusted using the Benjamini and Hochberg’s approach for controlling the false discovery rate. Those with an adjusted P-value below 0.05 were considered as differentially expressed genes (DEGs). Heatmaps were made using online tool Chiplot (https://www.chiplot.online).

### 2.4 Functional enrichment analysis

Functional categorization of the DEGs was performed by using Gene Ontology (GO) database and Kyoto Encyclopedia of Genes and Genomes (KEGG) database. GO enrichment analysis of DEGs was performed by using the BLAST2GO platform (Conesa et al., 2005) and the GOseq R package (Young et al., 2010). GO terms with corrected P value below 0.05 were considered significantly enriched. KEGG enrichment analysis of DEGs was performed by using the KEGG Automatic Annotation Server (Moriya et al., 2007) and KOBAS software (Mao et al., 2005). KEGG terms with false discovery rate (FDR) below 0.05 were considered significantly enriched.

### 2.5 Quantitative real-time PCR

In order to confirm the genes expression profiles resulting from high throughput RNA-seq analysis, we performed real-time PCR to analyze the expressions of the key genes from each pathway or functional group. The real-time PCR kit was from QIAGEN (208054, Germany). The primers used in real-time PCR were in the **Supplementary Materials** (**Supplementary File 2**). The reference genes used in real-time PCR were elongation factor 1-alpha. The relative expression quantification was calculated by the 2^-ΔΔCt^ method (Livak and Schmittgen, 2001).

## 3 Results

### 3.1 Overview of the transcriptomic response

There were 13509 genes annotated in the genome of *Dunaliella salina*. Among the 9537 genes annotated by the GO database, the number of differently regulated genes (DEGs) increased from 4142 at 0.5-hour under stress, to 4568 at 1-hour, and to 6577 at 2-hour, accounting for about 31%, 34%, and 49% of the total genes. The number of DEGs increased with the increasing of stress time. By using GO enrichment analysis, most of these differently regulated genes (DEGs) can be clustered into some biological processes. These biological processes reflect the responses during the first two hours of *Dunaliella salina* when confronting salt stress at a more global level, include transcription, protein synthesis, protein degradation, protein folding, protein modification, protein transport, cellular component organization, cell redox homeostasis, DNA repair, glycerol synthesis, energy metabolism, lipid metabolism, and ion homeostasis. Among them, protein folding, DNA repair, and cell redox homeostasis are the three eye-catching ones characterized by the nearly ubiquitous up-regulation of the genes involved in them. All the biological processes are analyzed and discussed in the following sections.

### 3.2 Biological processes being regulated under salt stress

#### 3.2.1 Transcription

##### 3.2.1.1 Regulation of transcription initiation

There were 33 genes encoding subunits of DNA-directed RNA polymerases (RNAP) annotated in the genome of *Dunaliella salina*. Twenty-two of them were differentially expressed, including the largest and catalytic core components of RNAP I, II and III (subunit rpa1, subunit 1, and subunit rpc1 respectively), the second largest components of RNAP II and III (subunit RPB2 and subunit RPC2), components of RNAP II, IV and V (subunit 11 and 12), and other subunits of RNAPs. The chloroplastic/mitochondrial specific RNA polymerase 2 was also included (**Supplementary File 1**: **Figure S1**).

Lots of transcription factors (TFs) were differentially expressed, including general transcription factors and gene specific transcription factors. The general TFs included subunits of RNA polymerase II transcription factor B, transcription factor IIH subunit 2, transcription termination factor MTERF9, mediator of RNA polymerase II transcription subunit 6, etc. The gene specific TFs, which regulate specific genes, included ethylene-responsive transcription factor AIL1, transcription factor VIP1, heat stress transcription factor A-1d, leucine zipper transcription factor-like protein 1, etc. (**Supplementary File 1**: **Figure S2**). Previous studies show that these TFs participated in variety of biologic processes including response to abiotic stresses (salt, osmotic, and heat stress), DNA repair, cell cycle, lipid accumulation, protein transport, etc. (**Table 1**).

**Table 1:** Differently expressed transcription factors and their reported function.

Gene ID	Name	Function
General TFs		
DsaChr060759	Mediator of RNA polymerase II transcription subunit 6	A coactivator involved in the transcription of RNAP II-dependent genes. 10.1128/MCB.17.8.4622
DsaChr090323	Transcription termination factor MTERF9, chloroplastic	Required for processing and steady-state levels of plastid transcripts. Involved in response to abiotic stresses. 10.1111/ppl.12307
DsaChr020919	General transcription factor IIH subunit 2	Component of TFIIH core complex, which is involved in DNA repair and transcription by RNAP II. (PubMed: 16623910).
DsaChr090639	Probable RNA polymerase II transcription factor B subunit 1-1	Component of TFIIH core complex, which is involved in DNA repair and transcription by RNAP II. PMID: 8194529
DsaChr160111	RNA polymerase II transcription factor B subunit 2	Component of TFIIH core complex, which is involved in DNA repair and transcription by RNAP II. PMID: 8194529
DsaChr050631	RNA polymerase II transcription factor B subunit 4	Component of TFIIH core complex, which is involved in DNA repair and transcription by RNAP II. PMID: 8194529
DsaChr030808	Transcription factor IIIB 90 kDa subunit	Involved in the transcription of tRNA, 10.1128/mcb.11.10.5181-5189.1991
Gene specific TFs		
DsaChr130238	Leucine zipper transcription factor-like protein 1	Response to salt stress, 10.1016/j.gene.2005.04.014
DsaChr010232	Transcription factor VIP1	Involved in osmosensory response, (PubMed: 25093810).
DsaChr030409	Heat stress transcription factor A-1d	High light and heat stress, 10.1093/pcp/pcr045
DsaChr120169	Nuclear transcription factor Y subunit C-2	Activated by Photooxidative Stress, 10.3390/ijms13033458
DsaChr040243	AP2-like ethylene-responsive transcription factor AIL1	Involved in biotic and abiotic stress, 10.3389/fpls.2019.00228
DsaChr100475	Nascent polypeptide-associated complex subunit beta	Play a central role as a proteostasis sensor, control translational activity in response to stress, 10.1038/emboj.2013.87
DsaChr030772	Transcription factor bHLH140	Involved DNA repair, 10.1093/bib/bbr042
DsaChr060567	Transcription factor Pur-alpha 1	Involved in cell cycle and DNA repair 10.4161/cc.8.3.7585
DsaChr050740	Paired amphipathic helix protein Sin3b	Acts as a transcriptional repressor, regulates cell cycle progression (PubMed: 22476904).
DsaChr070147	Transcription factor BOA	Transcription factor that is a critical component of the regulatory circuit of the circadian clock. 10.1105/tpc.111.084293
DsaChr060003	Transcription factor MYB1	Lipid accumulation, 10.1111/nph.18141
DsaChr110109	Ankyrin repeat domain-containing protein 26	Acts as a regulator of adipogenesis.10.1371/journal.pone.0038130
DsaChr060686	Ankyrin repeat domain-containing protein 50	Involved in protein transport (PubMed: 25278552).
DsaChr090477	Helicase-like transcription factor CHR27	Involved in transcriptional gene silencing. (PubMed: 25420628).
DsaChr040325	Protein BFR2	Involved in 18S ribosomal RNA processing, 10.1093/nar/gkt1293
DsaChr120929	Putative transcription elongation factor SPT5 homolog 1	Enhances transcription, 10.1128/MCB.00609-09
DsaChr030474	Transcription factor 25	May play a role in cell death control. 10.1016/j.bbrc.2006.02.187
DsaChr020321	Transcription factor A, mitochondrial	DNA packaging factor in mitochondria regulating mitochondria gene expression, 10.1073/pnas.1119738109
DsaChr050461	Transcription factor DIVARICATA	Involved in the dorsovental asymmetry of flowers. Promotes ventral identity, flower development, 10.1101/gad.221002.

##### 3.2.1.2 Pre-initiation regulation of transcription

Besides the TFs involved in regulations of transcription, some genes involved in chromatin remodeling and histone modification were also differentially expressed, including histone-lysine N-methyltransferases, histone acetyltransferases, CHD3-type chromatin-remodeling factor PICKLE, protein chromatin remodeling 20, bromodomain-containing protein 7, etc. The regulation of these genes reflects pre-initiation regulations of transcription which affect the binding of the core transcriptional machinery proteins to the core promoter sequence on the coding region of the DNA.

##### 3.2.1.3 Post-transcriptional regulation

Some genes involved in post-transcriptional regulations (RNA processing) were also differentially expressed, including pre-mRNA splicing factors, mRNA decapping enzymes, tRNA & rRNA methyltransferases, Zinc phosphodiesterases, Component of the SSU processome, RAP domain-containing protein, etc. These DEGs can be classified by function into groups of pre-mRNA splicing, mRNA decay, and tRNA and rRNA processing.

#### 3.2.2 Protein synthesis and degradation

##### 3.2.2.1 Protein synthesis

The GO term of peptide biosynthetic process was significantly enriched by DEGs. Lots of genes encoding ribosomal proteins were up-regulated, including chloroplast, mitochondrial, and cytosolic ribosomal protein genes (**Supplementary File 1**: **Figure S3**). Most of the 40S and 60S ribosomal proteins showed up-regulation at 0.5-hour and 1-hour under stress, and return to normal level at 2-hour under stress, on the other side, most of the 30S and 50S ribosomal proteins showed up-regulation at 2-hour of stress (**Supplementary File 1**: **Figure S3**). Lots of aminoacyl-tRNA ligases, translation initiation factors, and translation elongation factors were also up-regulated (**Supplementary File 1**: **Figure S4**). The up-regulation of these genes possibly reflect the accelerating of protein synthesis. The child term glycoprotein biosynthetic process was also enriched, subunits of dolichyldiphosphooligosaccharide-protein glycosyltransferase, Exostosin-like 2 & 3, and UDP-glucose:glycoprotein glucosyltransferase were up-regulated, which may reflect the regulation of glycoprotein synthesis.

##### 3.2.2.2 Protein degradation

On the other side, the GO term of ubiquitin-dependent protein catabolic process was enriched. Lots of ubiquitin-protein ligases, ubiquitin-conjugating enzymes (**Supplementary File 1**: **Figure S5**), and subunits of 26S proteasome were up-regulated (**Supplementary File 1**: **Figure S6**). The up-regulation of the genes possibly suggest the acceleration of protein degradation by ubiquitin proteasome system (UPS).

The accelerating of both protein synthesis and ubiquitin-dependent protein degradation possibly reflect the accelerating of protein dynamic change under salt stress.

#### 3.2.3 Protein folding

In cytosol, protein folding starts with the help of a first tier of ribosome-associated chaperones (mainly Hsp70 and Hsp40 which stabilize the nascent polypeptides) which form complex with ribosome called ribosome-associated complex (RAC), then a second tier of components including chaperonins and Hsp90 act downstream in completing the folding process (Vabulas et al., 2010). The ubiquitous up-regulation of Hsp40s (also called chaperone protein DnaJ), Hsp70s, and Hsp90s were observed (**Supplementary File 1**: **Figure S7**). The subunits of T-complex protein 1 (the group II chaperonin) and prefoldin were also up-regulated (**Supplementary File 1**: **Figure S7**).

In endoplasmic reticulum, protein folding participants include luminal binding proteins (BiPs) which bind to nascent polypeptides to prevent their aggregation, ER-localized DNAJ domain-containing proteins (ERdj proteins) which are BiP cochaperones that assist protein folding, calreticulin (CRT, folding apparatus) which sequester nascent glycoproteins to facilitate folding, UDP-glucose: glycoprotein glucosyltransferase (UGGT) which tells CRT if the glycoprotein needs another round of folding by adding a terminal glucose to the glycoprotein, and protein disulfide isomerases (PDIs) which catalyzes the breaking and reforming of disulfide bonds (Liu and Howell, 2016). These genes were all up-regulated except one ERdj gene (the other ERdj was up-regulated) (**Supplementary File 1**: **Figure S7**). Besides the genes mentioned above, there were as much as 37 genes encoding peptidyl-prolyl cis-trans isomoerases, only 3 of them do not showed up-regulation (**Supplementary File 1**: **Figure S8**). We speculate that the non-up-regulated genes possibly been up-regulated before 0.5-hour of stress. The expressional profiles of these genes suggest that protein folding was strongly enhanced under salt stress.

#### 3.2.4 Protein modification

Lots of DEGs were enriched in protein modification processes including phosphorylation, glycosylation, ubiquitination, lipidation, dephosphorylation, methylation, alkylation, acetylation, and mannosylation. Among these modifications, protein phosphorylation was the most eye-catching one with the largest number of DEGs involved, accounting for about 60% of the total DEGs of protein modification. A lot of protein kinases enriched in protein phosphorylation, included serine/threonine-protein kinases, cyclin-dependent kinases, calcium-dependent protein kinases, mitogen-activated protein kinases, subunits of cAMP-dependent protein kinases, etc. By GO enrichment analysis, these kinases participate in a variety of cellular biological processes including response to abiotic stress (including salt, osmotic, drought, oxidative, and cold stress), response to DNA damage and lead to cell cycle control, response to unfolded protein, RNA processing, carbohydrate metabolism, photosystem II regulating, microtubule organization, and chloroplast protein import (**Table 2**). These biological processes, except the response to abiotic stress which is a general expression, represent most of the aspects of this paper that are discussed or will be discussed in the following sections. Upon the whole, the regulation of these kinases suggests that most of the biological processes of *Dunaliella salina* in response to salt stress are carried out through the way of protein phosphorylation by kinases.

**Table 2:** Selected kinases and the related biological processes.

BP/Gene ID	Gene name	Reference (PMID)
Salt stress		
DsaChr020159	Calcium-dependent protein kinase 12	21883553
DsaChr060221	Serine/threonine-protein kinase AtPK2/AtPK19	29084871
Osmotic/drought stress		
DsaChr100125	Serine/threonine-protein kinase SRK2C	15561775
DsaChr010085	Serine/threonine-protein kinase SRK2E	30361234
Oxidative stress		
DsaChr030312	Mitogen-activated protein kinase kinase kinase ANP1	10717008
Cold stress		
DsaChr080233	CBL-interacting serine/threonine-protein kinase 7	21600398
DsaChr060221	Serine/threonine-protein kinase AtPK2/AtPK19	29084871
Response to unfolded protein		
DsaChr130345	Serine/threonine-protein kinase ppk4	23066505
DsaChr110405	Serine/threonine-protein kinase/endoribonuclease IRE1	11779464
DsaChr050352	Serine/threonine-protein kinase/endoribonuclease IRE1	22050533
DNA damage/cell cycle control		
DsaChr090603	Cyclin-dependent kinase 7	10024882
DsaChr060653	Serine/threonine-protein kinase ATM	12509526
DsaChr010826	Serine/threonine-protein kinase Nek1	20230784
DsaChr010422	Serine/threonine-protein kinase Nek4	22851694
RNA processing		
DsaChr100614	Serine/threonine-protein kinase SMG1	15175154
DsaChr020456	Serine/threonine-protein kinase RIO1	22072790
Microtubule/cytoskeleton organization		
DsaChr010549	Mitogen-activated protein kinase 4	20215588
DsaChr060318	Mitogen-activated protein kinase kinase kinase 11	12529434
DsaChr070114	Mitogen-activated protein kinase kinase kinase 2	20215588
DsaChr010906	Serine/threonine-protein kinase phg2	15194808
DsaChr120313	Serine/threonine-protein kinase RUNKEL	19268593
Carbohydrate metabolism		
DsaChr120587	SNF1-related protein kinase catalytic subunit alpha KIN10	17671505
Chloroplast protein import		
DsaChr110313	Serine/threonine-protein kinase STY17	17090544
DsaChr100019	Serine/threonine-protein kinase STY46	17090544
DsaChr020711	Serine/threonine-protein kinase STY8	17090544
Photosystem II regulating		
DsaChr050007	Serine/threonine-protein kinase STN8, chloroplastic	16040609
DsaChr130132	Serine/threonine-protein kinase stt7, chloroplastic	12624266

#### 3.2.5 Protein transport

There are two kinds of protein transport, co-translational transport (vesicle-mediated transport) and post-translational transport (non-vesicular transport) (Jungnickel et al., 1994). Compared to post-translational transport, co-translational transport is reported to involve in abiotic stress response (Wang et al., 2020), so we analyze only co-translational transport here. The co-translational transport pathway utilizes the signal recognition particle (SRP) to deliver proteins to the ER membrane while they are still being synthesized by ribosomes (Nyathi et al., 2013). On ER membrane with the help of Sec translocon (Sec61 complex), the nascent polypeptides enter the lumen of ER where they fold to their final conformation (Denks et al., 2014). Then the protein cargos can be transported from ER to Golgi, subsequentially from Golgi to Lysosome, to Plasma membrane, or to out of cell by vesicles. Protein cargos can also be transported from Golgi back to ER, or from Plasma membrane to Lysosome by vesicles (Paez Valencia et al., 2016). During the process, there are proteins and protein machineries work together to create vesicles from donor membranes, to target the vesicles to different destinations, and to fuse vesicles to target membranes (Paez Valencia et al., 2016). In *Dunaliella salina*, the genes encoding the proteins and the subunits of the machineries that involved in vesicle-mediate transport are summarized in **Table 3**. The SRP proteins including the SRP54 protein which carries the core function, the alpha and gamma subunits of sec61 translocon, and the subunits of coat proteins including COPI (required for vesicles formation from cis Golgi membrane), COPII (required for vesicles formation from ER membrane), and Clathrin (required for vesicles formation from trans Golgi membrane and plasma membrane), were up-regulated. The adaptor proteins and syntaxins were also up-regulated. The up-regulation of these genes suggests the enhancing of vesicle-mediated protein transport under salt stress. Lots of vacuolar protein sorting-associated proteins (VPS), including components of ESCRT-I, ESCRT-II, and ESCRT-III complex which involved in ubiquitin tagged proteins sorting and delivery to the endosome for degradation, were differently regulated. The SNARE proteins, which drive membrane fusion, were also differently regulated. Studies report that Sec31 (coat protein) (Chung et al., 2016), Sar1a (small GTPase, mediating COPII vesicle formation) (Zeng et al., 2021), Vps23 (component of ESCRT-I) (Yu et al., 2016), LIP5 (accessory protein mediating endosomal sorting) (Wang et al., 2015), and SKD1 (AAA-type ATPase mediating endosomal sorting) (Ho et al., 2010) participated in abiotic stress including drought and salt stress. The homologs of the five genes in *Dunaliella salina* were also up-regulated, which reflects the conservation of the response mechanism between the algae and *Arabidopsis thaliana* (**Table 3**).

**Table 3:** Genes participate in vesicle-mediate protein transport.

Gene ID	Gene name	log_2_(FoldChange)		
		0.5 h *VS* 0 h	1 h *VS* 0 h	2 h *VS* 0 h
SRP				
DsaChr110350	SRP 9 kDa	UP	UP	FALSE
DsaChr120098	SRP 14 kDa	UP	FALSE	FALSE
DsaChr110363	SRP 43 kDa	UP	UP	DOWN
DsaChr120629	SRP protein	UP	UP	UP
DsaChr100436	SRP subunit SRP72	FALSE	FALSE	UP
DsaChr020098	SRP 54 kDa protein 1	FALSE	FALSE	UP
Translocon				
DsaChr080295	Sec61 subunit alpha	UP	UP	UP
DsaChr070622	Sec61 subunit gamma	UP	FALSE	DOWN
COPI				
DsaChr010839	Coatomer subunit alpha-1	UP	UP	FALSE
DsaChr050350	Coatomer subunit zeta-2	FALSE	FALSE	UP
DsaChr080298	Coatomer subunit beta’-1	UP	UP	UP
DsaChr120756	Coatomer subunit beta-1	UP	UP	UP
DsaChr060027	Coatomer subunit gamma-2	UP	UP	UP
DsaChr040552	Coatomer subunit epsilon-1	UP	UP	FALSE
DsaChr050508	Coatomer subunit delta-1	UP	FALSE	UP
COPII				
DsaChr090410	Protein transport protein Sec24-like CEF	UP	UP	FALSE
DsaChr020331	Protein transport protein SEC13 homolog A	FALSE	FALSE	UP
DsaChr050599	Protein transport protein Sec24-like At3g07100	FALSE	UP	UP
DsaChr120331	Protein transport protein SEC31 homolog B	FALSE	UP	UP
DsaChr030638	GTP-binding protein SAR1A	FALSE	FALSE	UP
Clathrin				
DsaChr140276	Clathrin heavy chain 2	UP	UP	UP
DsaChr030331	Clathrin assembly protein At1g14910	UP	UP	DOWN
Adaptor				
DsaChr020052	Beta-adaptin-like protein A	UP	UP	DOWN
DsaChr121169	Beta-adaptin-like protein C	UP	UP	UP
DsaChr060511	AP-4 complex subunit epsilon	FALSE	FALSE	UP
DsaChr060511	AP-4 complex subunit epsilon	FALSE	FALSE	UP
DsaChr030303	AP-1 complex subunit gamma-2	FALSE	FALSE	UP
DsaChr050143	AP-2 complex subunit alpha-1	UP	UP	FALSE
Syntaxin				
DsaChr050130	Syntaxin-132	FALSE	FALSE	UP
DsaChr060051	Syntaxin-32	DOWN	DOWN	UP
DsaChr010717	Syntaxin-22	FALSE	FALSE	UP
DsaChr060183	Syntaxin-52	DOWN	DOWN	UP
VPS				
DsaChr010788	VPS29	UP	FALSE	DOWN
DsaChr010952	VPS26B	UP	UP	UP
DsaChr150146	VPS32 homolog 2	UP	UP	UP
DsaChr020689	VPS24 homolog 1	FALSE	DOWN	UP
DsaChr070483	VPS55 homolog	FALSE	DOWN	FALSE
DsaChr050295	VPS16 homolog	UP	UP	DOWN
DsaChr010029	VPS33A	UP	UP	DOWN
DsaChr140366	VPS28 homolog 2	DOWN	DOWN	FALSE
DsaChr080378	VPS18 homolog	DOWN	FALSE	DOWN
DsaChr010762	VPS35A	UP	UP	UP
DsaChr121235	VPS51	FALSE	FALSE	DOWN
DsaChr121048	VPS2 homolog 3	FALSE	FALSE	UP
DsaChr060632	VPS22 homolog 1	FALSE	DOWN	DOWN
DsaChr100474	VPS41 homolog	FALSE	UP	DOWN
DsaChr110426	VPS9A	FALSE	UP	FALSE
DsaChr110193	VPS13a	UP	UP	UP
DsaChr130018	VPS36	UP	UP	UP
DsaChr010386	VPS53 A	FALSE	FALSE	DOWN
DsaChr020102	VPS25	FALSE	DOWN	FALSE
DsaChr020245	VPS2 homolog 1	FALSE	FALSE	UP
DsaSca0152	VPS45 homolog	DOWN	FALSE	FALSE
DsaChr160069	VPS11 homolog	FALSE	UP	UP
DsaChr010996	VPS23	FALSE	FALSE	UP
DsaChr130098	LIP5	FALSE	UP	UP
DsaChr150326	SKD1	FALSE	FALSE	UP
Novelgene0862	ESCRT-related protein CHMP1B	FALSE	FALSE	UP
SNARE				
DsaChr110201	Vesicle transport v-SNARE 11	DOWN	FALSE	UP
DsaChr030800	Golgi SNAP receptor complex member 1-2	FALSE	DOWN	DOWN
DsaChr090438	R-SNARE, Tomsyn-like family	UP	UP	UP
DsaChr040011	Qb-SNARE protein, Sec20-family	UP	FALSE	UP
DsaChr110432	Qc-SNARE protein, USE1 family	FALSE	FALSE	DOWN
DsaChr121024	Qc-SNARE protein, Bet1/mBET1 family	FALSE	DOWN	FALSE
DsaChr070004	Vesicle-associated membrane protein 727	DOWN	FALSE	UP
DsaChr030249	Qc-SNARE protein, SFT1 family	FALSE	FALSE	DOWN
DsaChr070004	Vesicle-associated membrane protein 727	DOWN	FALSE	UP
DsaChr020111	Vesicle-associated membrane protein 714	DOWN	DOWN	UP
DsaChr030329	Vesicle transport protein GOT1	FALSE	FALSE	DOWN

“FALSE” means no significant up- or down-regulation, “UP” and “DOWN” means significant up-regulation and down-regulation respectively.

#### 3.2.6 Cellular component organization

When confronting salt stress, the *Dunaliella* cells decrease their volume quickly due to high extracellular osmotic pressure, and then gradually increase their cell volume by increasing the intracellular glycerol content to balance the osmotic pressure across plasma membrane. The whole process accomplishes in about 2 hours (Ben-Amotz and Avron, 1973). During the process, cellular component organization is a must. GO terms, such as membrane organization, endomembrane system organization, cytoskeleton organization, chromosome organization, protein-containing complex organization, etc., were enriched (**Supplementary File 1**: **Figure S9**). Here we only discuss membrane organization, endomembrane system organization, and cytoskeleton organization. The rest GO terms will not be discussed due to the limits of paper length. For membrane organization, it is well-known that autophagy, which was enriched in our study, involves organization of plasma membrane and reported to be induced by salt stress (Liu and Bassham, 2012). For endomembrane system organization, it is known that the vesicle-mediated protein trafficking is carried out by the way of endomembrane system organization as described in the section of protein transport. Besides membrane and endomembrane system organization, the other eye-catching GO term is cytoskeleton organization containing the most DEGs among the child terms of organelle organization ((**Supplementary File 1**: **Figure S9**).

#### 3.2.7 Glycerol synthesis and energy metabolism

##### 3.2.7.1 Glycerol synthesis

**Figure 1:**
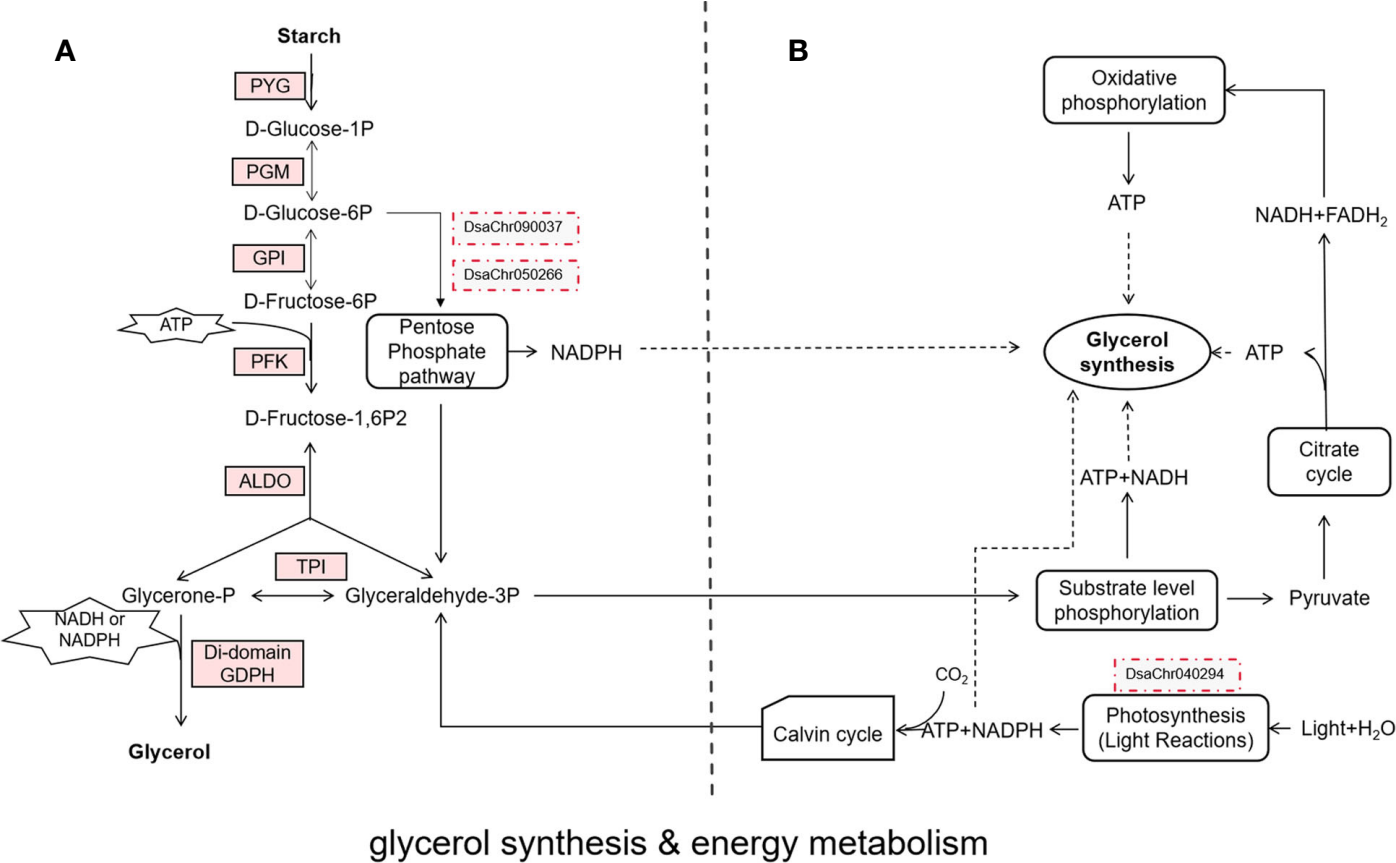
Glycerol synthesis & energy metabolism. **(A)** the glycerol synthesis pathway from starch to glycerol, the enzymes on the pathway are shown by rectangles, the up-regulated enzymes are indicated by light red background, the two red dotted boxes indicate the two key genes of pentose phosphate pathway confirmed by real-time PCR; **(B)** the possible sources (rounded rectangles) of ATP and reducing equivalents (NADH or NADPH) for glycerol synthesis, the dotted arrows indicate the ATP and reducing equivalents could be used for glycerol synthesis, the red dotted box indicates the gene confirmed by real-time PCR.

When confronting salt stress, *Dunaliella* cells rapidly shrink, then gradually recover its original volume by intracellular glycerol synthesis to balance the extracellular osmotic pressure (Ben-Amotz and Avron, 1973; Baek et al., 2011). The whole process finished in about 2 hours. Studies report that *Dunaliella* cells grown in 4 M NaCl contain about 8 M glycerol (Chitlaru and Pick, 1989; Oren, 2005). These studies indicate that rapid high-yield biosynthesis of glycerol is a special mechanism of *Dunaliella* to tolerate salt stress. In the last century, scientists suggested that the reserve starch pool is a carbon source of glycerol synthesis (Ben-Amotz and Avron, 1973), and proposed the glycerol cycle pathway which suggested that glycerol can be synthesized from and converted to DHAP (dihydroxyacetone phosphate or glycerone-phosphate) which is an intermediate metabolite of glycolysis (Haus and Wegmann, 1984) However a complete synthesis pathway from starch to glycerol was absent, here we propose a pathway from starch to glycerol based on our genomic data and the published papers (He et al., 2020b), the input of energy and reducing equivalents are also indicated (**Figure 1A**). The expressions of the enzymes were all up-regulated, the key enzymes are PYG, PFK, and the di-domain GPDH, which catalyze the three irreversible reactions.

Study also showed that photosynthesis is also a carbon source of glycerol synthesis (Goyal, 2007). In our study, the up-regulated genes enriched in the term photosynthesis included photosystem I reaction center subunits, photosystem II reaction center proteins, chlorophyII a-b binding proteins, oxygen-evolving enhancer proteins, PsbP domain-containing proteins, thylakoid luminal proteins, etc. We can see most of the genes showed a quick up-regulation of expression at 0.5 and 1-hour under stress followed by a down-regulation of expression at 2-hour under stress (**Supplementary File 1**: **Figure S10**). The expressional profiles of these genes suggest that photosynthesis was transiently up-regulated by salt stress.

##### 3.2.7.2 Energy metabolism

Study report that photosynthesis and reserved starch are the carbon source of glycerol synthesis, and reserved starch is the main source (Goyal, 2007). From the glycerol synthesis pathway that we proposed, synthesis of one molecule of glycerol needs 0.5 molecule of ATP and one molecule of NADH or NADPH (reducing equivalents) (**Figure 1A**). So, synthesis of large amount of glycerol needs large amount of ATP and reducing equivalents. Where do these ATP and reducing equivalents come from the GO enrichment analysis, glycolysis, TCA cycle and oxidative phosphorylation, which can generate ATP or NADH or both of them, were enriched. It is worth noting that pentose phosphate pathway (PPP) was also enriched. Glucose-6-phosphate dehydrogenase, the rate-controlling enzyme of PPP, was significantly up-regulated (Stryer and Co, 1995). 6-phosphogluconate dehydrogenase, the second NADPH producing enzyme in PPP, was also significantly up-regulated. Since generation of NADPH is one of the outcomes of PPP, the up-regulation of PPP could be a source of reducing equivalents for glycerol synthesis. From the starch catabolism pathway (**Figure 1A**), the carbon flux branches at the point of fructose 1, 6-biphosphate, some carbon goes to glycerol synthesis, the other goes to TCA cycle and oxidative phosphorylation to generate NADH and ATP which can be used for glycerol synthesis. Taking together, it seems that the large amount of ATP and reducing equivalents needed for glycerol synthesis could be provided by starch catabolism through glycolysis, pentose phosphate pathway, TCA cycle and oxidative phosphorylation (**Figure 1B**).

#### 3.2.8 Cell redox homeostasis

Efficient flux of electrons is vital for photosynthesis and respiration of plant cells. Efficient flux of electrons also means that the oxidized and reduced forms of electron carriers in electron transport chains must be balanced, which is called redox homeostasis (Foyer and Noctor, 2005). However, salt stress can disrupt the balance and induce the rise of reactive oxygen species (ROS) and lead to oxidative stress. To survive, organisms need to regain cell redox homeostasis by getting the excessive ROS reduced by small molecule antioxidants, such as ascorbate, glutathione (GSH), and tocopherol. The genes involved in regeneration of these antioxidants, such as monodehydroascorbate reductase and glutathione reductase, were mostly up-regulated at 0.5-hour under stress (**Supplementary File 1**: **Figure S11**). Another type of antioxidants is antioxidative proteins, such as thioredoxins, glutaredoxins, and peroxiredoxins. Most of the genes encoding antioxidative proteins were up-regulated at 0.5-hour under stress (**Supplementary File 1**: **Figure S11**). The genes involved in regeneration of these antioxidative proteins, such as thioredoxin reductase and methionine sulfoxide reductase, were also up-regulated at 0.5-hour under stress. Taken together, the genes involved in cell redox homeostasis responded quickly and strongly to salt stress by up-regulation of their expressions.

#### 3.2.9 DNA repair

Salt stress induces the rise of ROS. ROS have strong oxidizing potential and lead to DNA damage. When DNA damage happens, cells stop cell cycle and activate DNA repair mechanisms to repair DNA. If DNA repairs are successful, cell cycle is reactivated. On the contrary, if the repairs fail and DNA lesions accumulate, cells undergo apoptosis (Hu et al., 2016) From this perspective, the strength of ability of DNA repair influences the degree of salt tolerance of an organism. Although we know that *Dunaliella salina* is extremely salt tolerant, it was still astonishing to see that so many genes involved in DNA repair were up-regulated by salt stress. The DEGs included DNA repair proteins, DNA repair ligases, DNA repair helicases, endonucleases, glycosylases, DNA polymerases, replication factor C subunits, recombinases, and DNA methyltransferases, etc. Most of these genes were up-regulated at 0.5-hour and 1-hour under stress (**Supplementary File 1**: **Figure S12**).

#### 3.2.10 Lipid metabolism

In general, the up-regulation of the key genes in the biosynthetic pathways indicates the acceleration of *de novo* fatty acid synthesis and TAG synthesis under salt stress. The up-regulation of the desaturases indicates that the fatty acids and lipids undergo desaturation.

##### 3.2.10.1 Fatty acid metabolism

**Figure 2:**
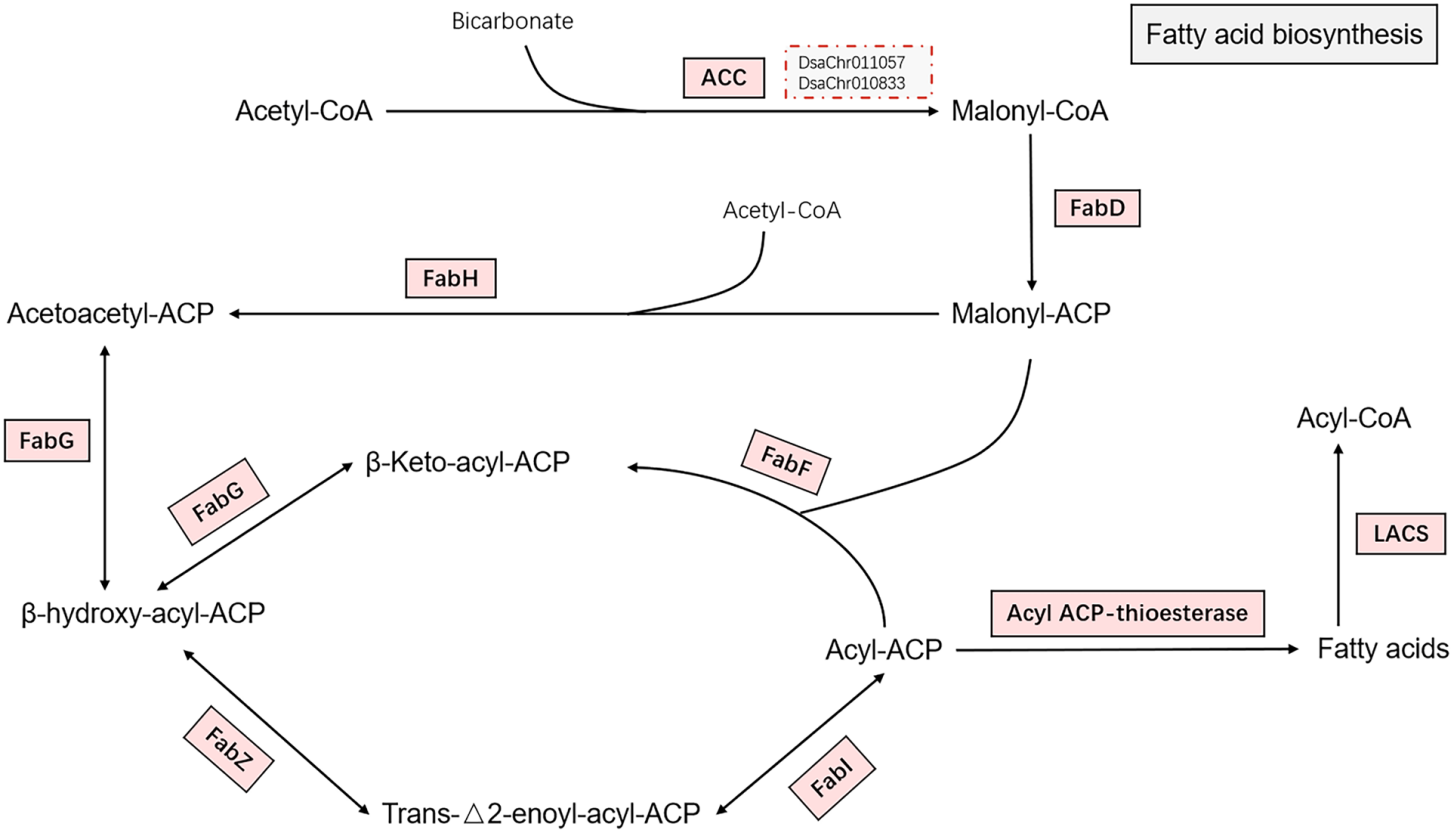
Fatty acid biosynthesis. The pathway of fatty acid biosynthesis from acetyl-CoA to fatty acids are shown in the figure. Enzymes catalyzing the reactions on the pathway are shown by rectangles. The enzymes were all up-regulated as indicated by light red background. The two red dotted boxes indicate the two key genes (biotin carboxylase and acetyl-CoA carboxylase) confirmed by real-time PCR. The full name of the enzymes are acetyl-CoA carboxylase (ACC), malonyl-CoA-ACP transacylase (FabD), 3-ketoacyl-ACP synthase II and III (FabF and FabH), 3-oxoacyl-ACP reductase (FabG), 3-hydroxyacyl-ACP dehydratase (FabZ), and enoyl-ACP reductase (FabI), acyl-protein thioesterase, and long chain acyl-CoA synthetase (LACS).

**Figure 3:**
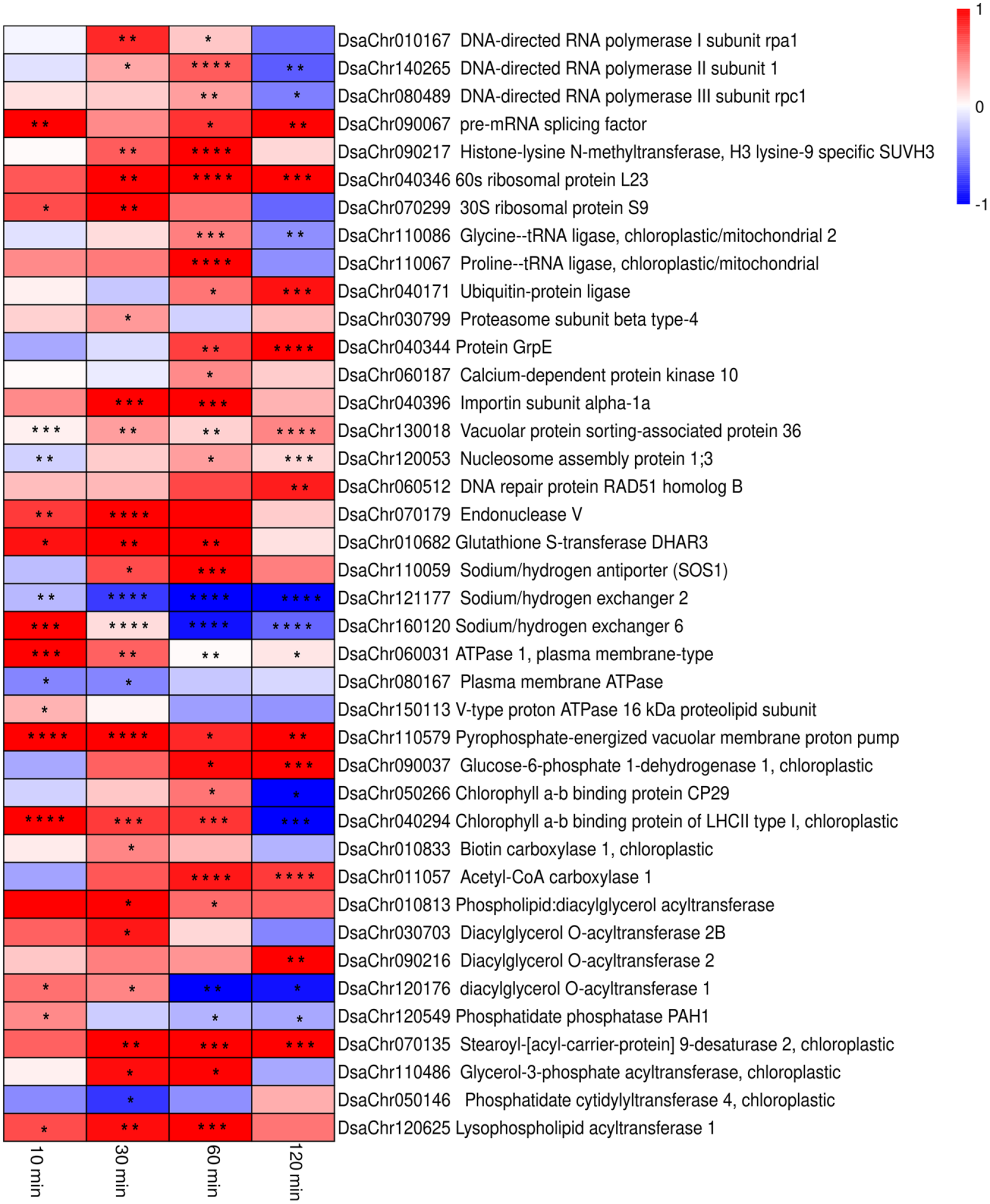
Heatmap of the expressions from real-time PCR of the selected key genes in the biological processes. The colors from blue to red represent the gene expression values from low to high. Values of log_2_ (Fold change) are used to generate the heatmap. The significance is indicated by asterisks in the heatmap (^*^ *P*<0.05,^**^ *P*< 0.01, ^***^ *P*< 0.001,^****^ *P *< 0.0001).

The chloroplastic acetyl-CoA carboxylase (heteromeric ACC) catalyzing the irreversible carboxylation of acetyl-CoA to produce malonyl-CoA, which is involved in *de novo* fatty acid synthesis in plants (Konishi et al., 1996), is composed of four independent polypeptides: biotin carboxyl carrier protein (BCCP), biotin carboxylase (BC), α carboxyl transferase, and β carboxyl transferase. The four subunits were all significantly up-regulated at 0.5-hour and 1-hour under salt stress. The cytosolic acetyl-CoA carboxylase (homomeric ACC) was also significantly up-regulated. The malonyl-CoA-ACP transacylase (FabD) and the other subunits of type II fatty acid synthase (FAS) including 3-ketoacyl-ACP synthase II and III (KAS II or FabF and KAS III or FabH), 3-oxoacyl-ACP reductase (FabG), 3-hydroxyacyl-ACP dehydratase (FabZ), and enoyl-ACP reductase (FabI), were all significantly up-regulated at 0.5-hour and 1-hour under salt stress (**Figure 2**). The expression of the biotin carboxylase and cytosolic acetyl-CoA carboxylase were confirmed by real-time PCR (**Figure 3**; **Supplementary File 2**). The up-regulation of these genes reflects the acceleration of *de novo* fatty acid synthesis under salt stress. Acyl-protein thioesterases and long chain acyl-CoA synthetases were also up-regulated, which possibly suggest the synthesized acyl-CoA could be used for lipid synthesis (Ohlrogge and Browse, 1995).

##### 3.2.10.2 Glycerolipid metabolism

**Figure 4:**
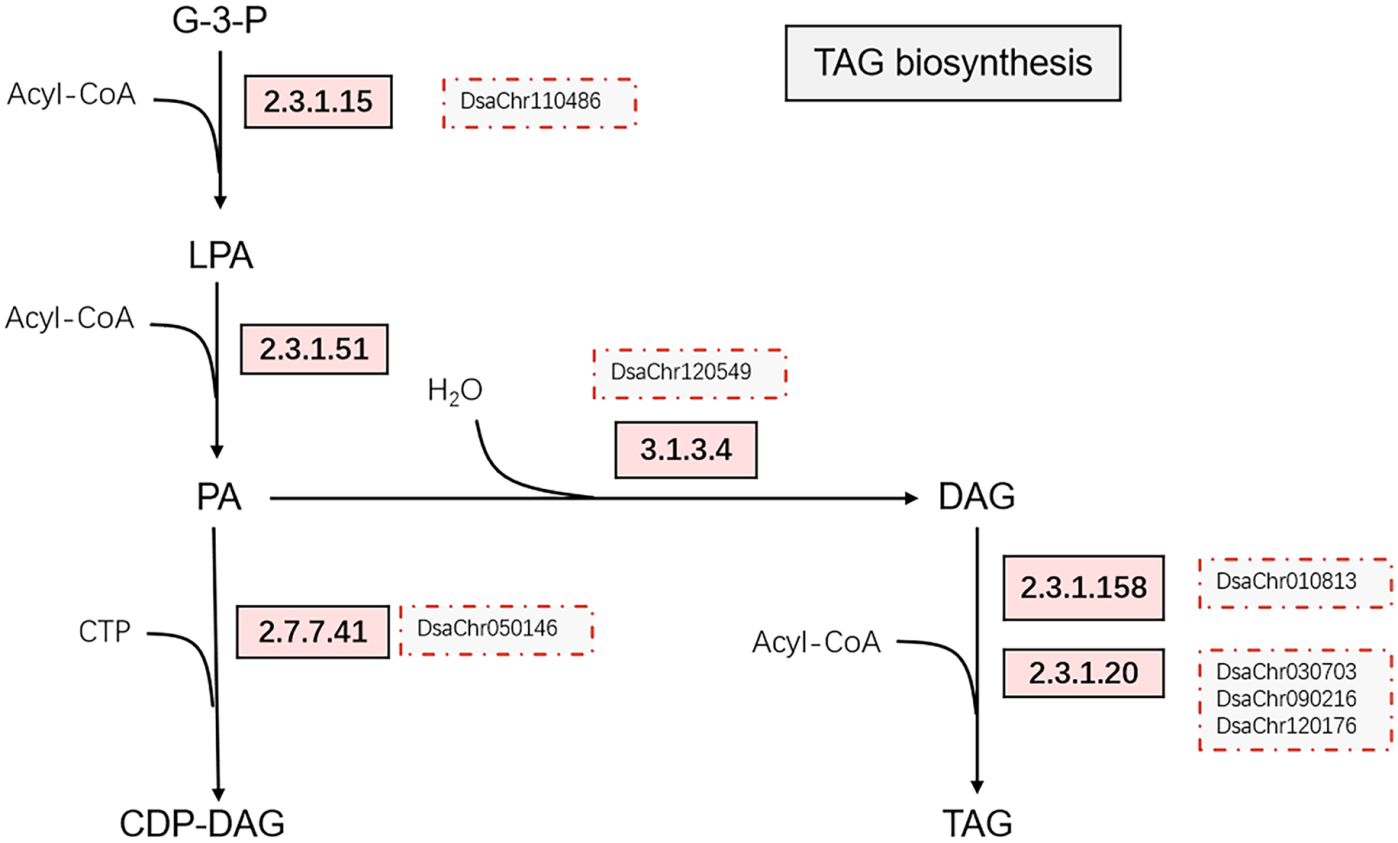
Simplified triacylglycerol biosynthesis pathway. The up-regulated enzymes catalyzing the reactions on the pathway are indicated by rectangles with light red background, the red dotted boxes indicate the genes confirmed by real-time PCR. The enzymes are glycerol-3-phosphate acyltransferase [2.3.1.15], 1-acylglycerol-3-phosphate O-acyltransferase [2.3.1.51], phosphatidate phosphatase [3.1.3.4], diacylglycerol O-acyltransferases [2.3.1.20], phospholipid:diacylglycerol acyltransferase [2.3.1.158], and phosphatidate cytidylyltransferases [2.7.7.41].

Glycerol-3-phosphate acyltransferase (EC: 2.3.1.15) and 1-acylglycerol-3-phosphate O-acyltransferase (EC: 2.3.1.51) were up-regulated at 0.5-hour under stress, which possibly suggest the acceleration of phosphatidic acid (PA) synthesis. The up-regulation of phosphatidate phosphatase (EC: 3.1.3.4) was detected at 10-min under stress by qPCR (**Figure 3**; **Supplementary File 2**). The up-regulation of the chloroplastic lipid phosphate phosphatase 3, which may exhibit phosphatidate phosphatase activity (Pierrugues et al., 2001; Nakamura et al., 2007), was also observed at 2-hour under stress. The expression of diacylglycerol O-acyltransferases (EC: 2.3.1.20) and phospholipid:diacylglycerol acyltransferase (EC: 2.3.1.158) were up-regulated at 10-min and 30-min under stress (**Figures 3**, **4**). Taken together, the expression profiles of these genes possibly suggest the acceleration of triacylglycerol (TAG) synthesis under stress.

Many desaturases were up-regulated under salt stress, including stearoyl-[acyl-carrier-protein] 9-desaturase (EC: 1.14.19.2), acyl-lipid omega-3 desaturase (chloroplastic, EC: 1.14.19.25), Delta12 fatty acid desaturase (EC: 1.14.19.6), Delta7-sterol 5(6)-desaturase (EC: 1.14.19.20), Palmitoyl-monogalactosyldiacylglycerol delta-7 desaturase (chloroplastic, EC: 1.14.19.42), Acyl-lipid (7-3)-desaturase (chloroplastic, EC: 1.14.19.31), and acyl-lipid omega-3 desaturase (chloroplastic, EC: 1.14.19.25) (**Supplementary File 1**: **Figure S13**). Upon the whole, the up-regulation of these desaturases suggest the desaturation of lipids under salt stress.

#### 3.2.11 Ion homeostasis

Studies report that Na^+^/H^+^ antiporters (or exchangers) located on plasma membrane are responsible for sodium ion extrusion, while Na^+^/H^+^ exchangers located on vacuolar membrane are for sodium ion sequestration in vacuole (Zhu, 2003). There is one gene, named as *DsaChr110059* and showing sequence similarity with Na^+^/H^+^ antiporter 7 of *Arabidopsis thaliana* (SOS1) which was reported to be responsible for Na^+^ and Li^+^ extrusion across plasma membrane (Wu et al., 1996; Shi et al., 2000), was significantly up-regulated at 0.5-hour and 1-hour under stress (**Figure 3**; **Supplementary File 2**). There are two genes, named as *DsaChr121177* and *DsaChr160120*, that show sequence similarities with Na^+^/H^+^ exchanger 2 and 6 of *Arabidopsis thaliana*, respectively, which are reported to be involved in vacuolar sodium ion compartmentalization (Yokoi et al., 2002; Bassil et al., 2011). Real-time PCR show that *DsaChr160120* was up-regulated at 10-min under stress, but *DsaChr121177* was not up-regulated (**Figure 3**; **Supplementary File 2**).

Studies also report that plasma membrane-type and vacuolar-type proton ATPase are responsible for creating proton gradient across plasma membrane and vacuolar membrane respectively, which could benefit Na^+^ efflux and sequestration by Na^+^/H^+^ antiporters and Na^+^/H^+^ exchangers (Yang and Guo, 2018). There are two genes, named as *DsaChr060031* and *DsaChr080167*, which show sequence similarities with plasma membrane ATPase 1 of *Arabidopsis thaliana* and plasma membrane ATPase of *Dunaliella bioculata*, respectively. *DsaChr060031* showed up-regulation at 10-min and 30-min under stress, but *DsaChr080167* didn’t show up-regulation (**Figure 3**; **Supplementary File 2**). There are 13 genes encoding subunits of vacuolar-type proton ATPase, 5 of them showed significant up-regulation. Besides the vacuolar-type proton ATPases, a gene encoding pyrophosphate-energized vacuolar membrane proton pump which also contributes to the proton gradient across vacuolar membrane showed up-regulation (Segami et al., 2018). The up-regulation of the vacuolar-type proton ATPases and the pyrophosphate-energized vacuolar membrane proton pump would lead to acidation of the vacuoles and benefit for Na^+^ sequestration.

Taken together, We did saw the up-regulation of a plasma membrane-type Na^+^/H^+^ antiporter (*DsaChr110059*), which shows sequence similarity with the well-studied SOS1 of *Arabidopsis thaliana* which was the main player of Na^+^ extrusion under salt stress (Zhu, 2003). We also saw the up-regulation of a vacuolar-type Na^+^/H^+^ exchanger (*DsaChr160120*) together with the up-regulation of some of the subunits of vacuolar-type proton ATPase and the up-regulation of a pyrophosphate-energized vacuolar membrane proton pump.

## 4 Discussion

This research gives a more comprehensive overview of how *Dunaliella* cells response to salt stress than the previous published papers do (Li et al., 2019; Lv et al., 2021; Panahi and Hejazi, 2021) due to high quality genomic data of this algae and appropriated stress time course setting. From the point of evolution, all plant cells, including higher plants, lower plants, and green algae, have similar cellular structure and respond to environmental stress similarly (Chapin, 1991), so this is also an overview of how photosynthetic cells response to salt stress. Although this overview is only at transcriptomic level, there are published papers to support most of the biological processes being discussed in this research, which also suggests that the regulations of the biological processes are conserved mechanisms of plants in response to salt stress.

The basic way of a plant cell to response to environmental changes is the regulation of gene expression. Besides the expressional regulations of the core components of transcription machineries, lots of the TFs were regulated in response to salt stress, many of them were reported to participate in abiotic stress response (**Table 1**). Moreover, the regulations at pre-initiation of transcription and post-transcriptional levels were also indicated by the DEGs, some of the DEGs were reported to be involved in salt tolerance of *Arabidopsis thaliana*, such as histone-lysine N-methyltransferase, histone acetyltransferase GCN5, and pre-mRNA splicing factors (Baek et al., 2011; Zheng et al., 2019).

Gene expression includes not only the transcription process, but also the translation process. The functional enrichment analysis of the DEGs indicates that the protein synthesis process was enhanced. This is reasonable because cells need to synthesize new proteins to adapt to salt stress. There are studies reported the association of glycoprotein with salt tolerance in *Arabidopsis* (Frank et al., 2008; Kang et al., 2008; Nagashima et al., 2018). What is interesting is that protein degradation was also enriched. In the last century, scientists found that mammal cells exhibit increased rates of proteolysis following exposure to oxidative stress (Pacifici et al., 1989; Grune et al., 1997)and proposed that ubiquitin proteasome system (UPS) is a major way for protein degradation (Hershko and Ciechanover, 1992). In plants, scientists found many homologs of mammal UPS, and suggested that UPS is also a major way for protein degradation (Smalle and Vierstra, 2004), however published works that report protein degradation induced by abiotic stress are rare (Ferguson et al., 1990; Neelam and Subramanyam, 2013). Here our data show that lots of ubiquitin-protein ligases, ubiquitin-conjugating enzymes, and subunits of 26S proteasome were up-regulated under salt stress, which indicate that UPS plays an important role in protein degradation under salt stress.

The protein folding process accompanies the translation process. Salt stress disrupts protein folding in ER and lead to ER stress. ER stress activates signal transduction pathway to up-regulate the expression of genes that aid in protein folding, such as chaperones, peptidyl prolyl isomerases, prefoldins, etc. (Liu and Howell, 2016). We saw the nearly ubiquitous up-regulation of the genes involved in protein folding, which indicates the process was strongly enhanced. However, reports of genetic engineering of protein folding to increase salt tolerance are rare (Wang et al., 2004). There are lots of molecular chaperones and enzymes that aid in protein folding, it is not likely to randomly choose a target that will lead to significant improvement of salt tolerance.

In eukaryotic cells, many newly synthesized proteins go to the post-translational modification process to become functional forms. There are two kinds of protein modifications that obviously related to salt stress response, one is ubiquitination, as mentioned above, the misfolded or damaged proteins must be attached the tag of ubiquitin before degradation. The other is protein phosphorylation, protein phosphorylation usually plays important role in signal transduction of stress sensing and response. To modulating signal transduction of salt stress response is a hopeful way to increase salt tolerance by changing phosphorylation state of key component of signal cascade. For example, the activity of Na^+^/H^+^ antiporter (SOS1) in salt overly sensitive (SOS) pathway is control by phosphorylation (Shi et al., 2000; Dittmore et al., 2016). Phosphorylation can activates SOS1 and might increase Na^+^ exclusion and lead to improvement of salt tolerance. Besides ubiquitination and phosphorylation, reactive oxygen species (ROS) and reactive nitrogen species (RNS) were reported to regulate ion transporters by protein modifications. These modifications include cysteine oxidation, methionine oxidation, cysteine S-nitrosylation, and tyrosine nitration (Nieves-Cordones et al., 2019; Sandalio et al., 2023). Salt stress can induce the rise of ROS and probably RNS, which could lead to oxidation or nitration of the residues of ion transporters and regulate their activities. Garcia-Mata et al. (Garcia-Mata et al., 2010) demonstrated that the residue Cys168 of SKOR K^+^ Channel was essential for sensitivity to H_2_O_2_. However more experiments on other transporters at protein molecular or structural level are still needed to show how ROS and RNS regulate the activities of transporters through residues oxidation or nitration in response to salt stress.

Since proteins are synthesized far away from their functional sites, protein transport is a fundamental cellular process that can be regulated in response to environmental changes. The vesicle-mediated protein transport involves ER as the very important departure station of protein cargos. Salt stress disrupt protein folding in ER, leading to accumulation of unfolded or misfolded proteins in ER which is also called ER stress (Liu et al., 2010). ER needs to activate signal transduction pathway to up-regulate the expression of genes that aid in protein folding, such as chaperones, and to send the unfolded proteins cargos to degradation. This is how salt stress intersects protein transport. By loss of function mutation techniques, researchers identified some participators of vesicle-mediated protein transport involved in salt stress response. Loss of function mutants were sensitive to salt stress compare to wild type plants (Wang et al., 2020). Although we know that protein transport is tightly linked to salt stress response, the underlying mechanisms are largely unknown.

Cellular component organization plays an essential role in a variety of biological processes, such as vesicle-mediated protein transport, cytoskeleton dynamics, autophagy, etc. Autophagy is a bulk degradation pathway that is essential for cell survival under nutrient-limiting conditions (Levine and Klionsky, 2004). Later scientists found that autophagy helps maintain cellular homeostasis under oxidative stress by degradation and recycling of the oxidized proteins and other cellular components (Bassham, 2007). Studies showed directly that salt stress induced autophagy of *Arabidopsis* within 30 min and mutants defective in autophagy were sensitive to salt stress (Leshem et al., 2007; Luo et al., 2017). These studies indicate that autophagy plays an important role in plant response to salt stress. Leshem et al. also reported that phosphoinositide signaling pathway is involved in autophagy under salt stress (Leshem et al., 2007). Considering that phosphoinositide signaling is also involved in the organization of cytoskeleton (Yin and Janmey, 2003) and the regulation of vesicle trafficking (Roth, 2004), we speculate that these biological processes might be coordinated by phosphoinositide signaling pathway in response to salt stress. However more work needed to elucidate how they can be regulated.

Salt stress induces rise of ROS and leads to disruption of redox homeostasis which is vital for proper cellular function. To survive, cells need to regain redox homeostasis by removing the excessive ROS. We saw most of the genes involved in cellular redox homeostasis were up-regulated at 0.5-hour under stress and last to 2-hour under stress, which indicates that the process was quickly and strongly enhanced. The quick and strong type of response of these genes also reflects the strong ability of *Dunaliella salina* to maintain cell redox homeostasis under salt stress. Overexpression of the genes encoding antioxidative enzymes led to improved salt tolerance (Agarwal et al., 2013; Wang et al., 2020), which indicates the importance of redox homeostasis in response to salt stress.

The rise in ROS also brings another serious consequence that is DNA damage. When DNA damage happens, cells activate DNA repair mechanisms to repair DNA (Hu et al., 2016). We saw that most of the genes involved in DNA repair were up-regulated at 0.5-hour and 1-hour under stress, which reflects the quick and strong response of *Dunaliella salina* to maintain DNA correctness under salt stress. *Dunaliella salina* can survive in medium containing NaCl as high as 5.5 M, so it is unexpected that salt stress of 2.5 M NaCl would cause such a strong response. On the other side, the strong response of DNA repair possibly reflects the high efficiency of DNA repair, which may be a major reason for the algae to survive in hypersaline environment.

There are studies reported that salt stress induced synthesis of fatty acid and total lipids in some microalgae (Takagi et al., 2006; El Arroussi et al., 2015; Pandit et al., 2017), and desaturation of fatty acids were reported in a few species(López-Pérez et al., 2009; Harrathi et al., 2011; Sui and Han, 2014). In our study, we see the up-regulation of the key enzymes ACCs (both the chloroplastic form and the cytosolic form) of the *de novo* fatty acid synthesis pathway and the enzymes in TAG synthesis pathway, which indicates the synthesis of fatty acids and TAG. The TAG contents were indeed increased (our unpublished data) under salt stress. We also see the up-regulation of the fatty acids desaturases, which indicates the lipids undergo desaturation under salt stress. Our data are in consistent with the previous studies. Moreover, Overexpression of *des*A encoding Δ12 acyl-lipid desaturase in *Synechococcus* led to improved salt tolerance(Allakhverdiev et al., 2001). Although these studies indicate that lipid metabolism is relevant to salt stress response, the underlying mechanism is unclear. Scientists speculate that unsaturated fatty acids can act as modulators of cellular membrane to increasing the fluidity of membrane under salt stress (He and Ding, 2020), and they also suggested that glycerolipids can work as carbon and energy reservoir under stress (He and Ding, 2020).

Besides the above discussed aspects, salt stress has two direct impacts on plant cells, one is that high salt content can alter the osmotic potential across plasma membrane and results in dehydration of plasma. The rapid high-yield biosynthesis of glycerol is a special mechanism of *Dunaliella* to cope with osmotic stress (Chitlaru and Pick, 1989; Oren, 2005). Based on our data, we propose a pathway from starch to glycerol, and suggest that PYG, PFK, and the di-domain GPDH are the three rate-limiting enzymes of the glycerol synthesis pathway. Our published paper showed that the di-domain GPDH can convert DHAP directly to glycerol whereas a separate phosphatase protein is required for this conversion process in most organisms, and the homotetramer structure of di-domain GPDH likely contributes to the rapid biosynthesis of glycerol (He et al., 2020b).

The other impact is that under high salt content, Na^+^ enters cell more easily, which will disrupt the intrinsic homeostasis of high concentration of K^+^ and low concentration of Na^+^, which is vital for diverse cellular processes. We find in *Dunaliella* the up-regulation of the homolog of SOS1 of *Arabidopsis* which is the main player of Na^+^ extrusion under salt stress (Zhu, 2003). We also find the up-regulation of a vacuolar-type Na^+^/H^+^ exchanger, some of the subunits of vacuolar-type proton ATPase, and a pyrophosphate-energized vacuolar membrane proton pump. The up-regulation of these genes is consistent with the expressions of their homologs in *Arabidopsis thaliana* under salt stress (Wu et al., 1996; Shi et al., 2000; Yokoi et al., 2002), which might suggest that these genes are responsible for ion homeostasis.

Finally, what light on the expression profile is the nearly ubiquitous up-regulation of the genes involved in protein folding, DNA repair, and cell redox homeostasis, which suggests that the three biological processes were strongly enhanced under salt stress, in reverse, it also implicates that we can improve plants salt tolerance by enhancing the activities of the three biological processes. Modulating the signal transduction pathways that control the three biological processes might be a promising way to improve salt tolerance. Considering that salt tolerance is a high energy and resource costing process, fine tuning is very important to achieve the balance between growth rate and degree of salt tolerance.

## 5 Conclusion

**Figure 5:**
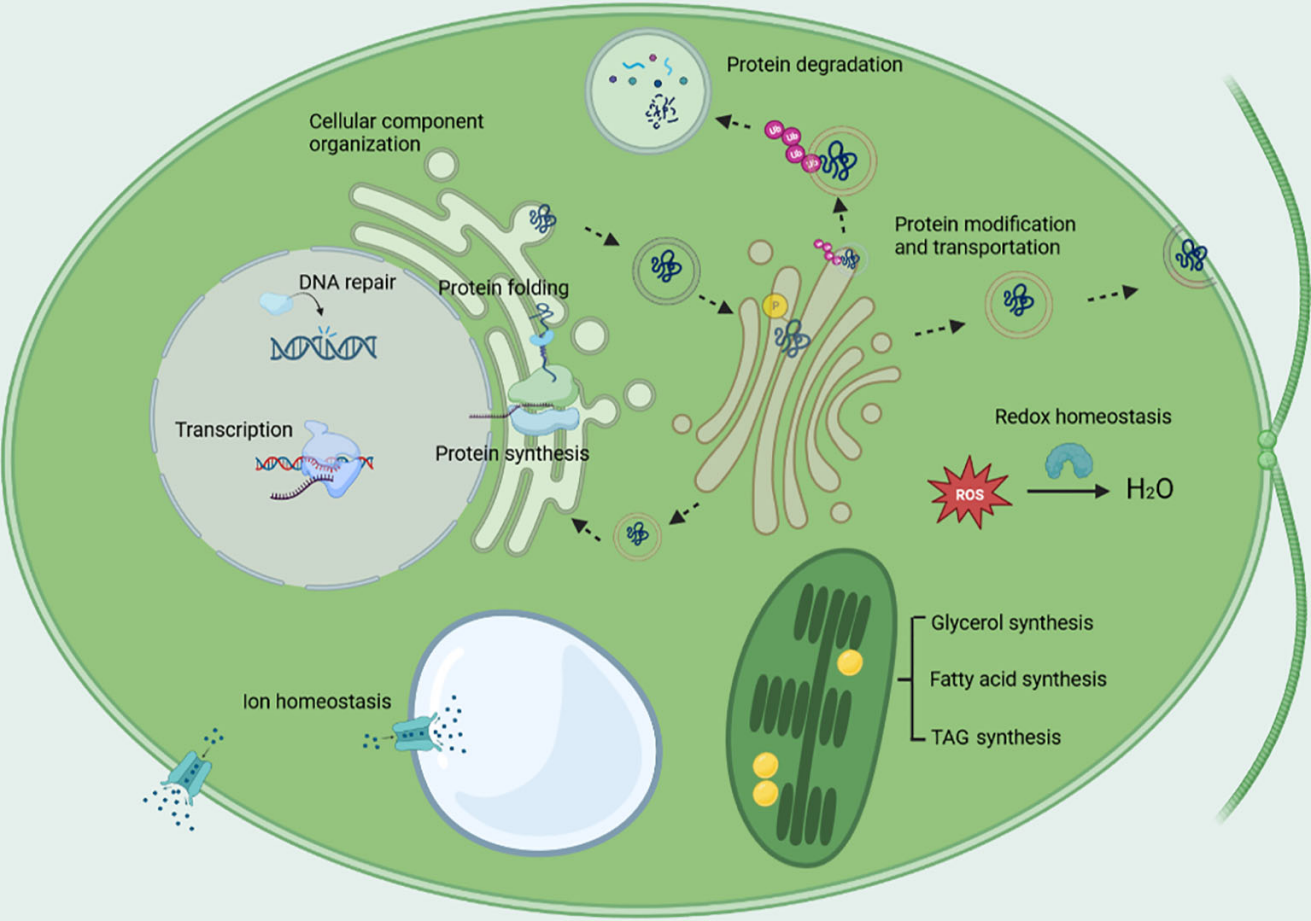
The major biological processes being regulated under salt stress. The biological processes include transcription, protein synthesis, protein degradation, protein folding, protein modification, protein transport, cellular component organization, cell redox homeostasis, DNA repair, glycerol synthesis, energy metabolism, lipid metabolism, and ion homeostasis.

The major cellular biological processes that being regulated in response to salt stress, are shown in **Figure 5**. The GO term of transcription was enriched, the regulation of transcription occurred at pre-initiation, initiation, and post transcription level; protein synthesis and protein degradation were enhanced at the same time, which implicates the acceleration of protein turnover; protein modification was enriched included a lot of protein kinases, which implicates protein phosphorylation plays an important role in response to salt stress; protein transport was enriched, the vesicle-mediated protein transport was enhanced; cellular component organization, which plays an essential role in vesicle-mediated protein transport, cytoskeleton dynamics, autophagy, was enriched; glycerol synthesis was enhanced, the energy for glycerol synthesis might be provided by starch catabolism through glycolysis, pentose phosphate pathway, TCA cycle and oxidative phosphorylation; the synthesis of fatty acids and TAG were enhanced, while desaturation of lipids was also enhanced; the up-regulation of the plasma membrane-type Na^+^/H^+^ antiporter and the vacuolar-type Na^+^/H^+^ exchanger together with the vacuolar-type proton ATPase and the pyrophosphate-energized vacuolar membrane proton pump might aid in ion homeostasis; protein folding, cell redox homeostasis, and DNA repair were greatly enhanced as indicated by the nearly ubiquitous up-regulation of the genes involved in the three biological processes, which may confer the algae important mechanisms to survive under salt stress, also implicates that enhancing the activities of the three biological processes are promising strategies to improve crop salt tolerance.

## Data availability statement

The datasets presented in this study can be found in online repositories. The names of the repository/repositories and accession number(s) can be found below: Sequence Read Archive under accession number SRR8552788, and SRR8543799 to SRR8543810. The genome data is deposited in the Genbank under accession number JAVFKU000000000.

## Author contributions

BZ: Data curation, Formal Analysis, Validation, Writing – review & editing. CD: Data curation, Formal Analysis, Validation, Writing – review & editing. SW: Formal Analysis, Investigation, Writing – review & editing. QD: Formal Analysis, Investigation, Writing – review & editing. YC: Formal Analysis, Validation, Writing – review & editing. ZB: Formal Analysis, Validation, Writing – review & editing. AH: Data curation, Formal Analysis, Validation, Writing – review & editing. QZ: Conceptualization, Supervision, Writing – review & editing, Writing – original draft. QH: Conceptualization, Funding acquisition, Methodology, Resources, Supervision, Writing – original draft, Writing – review & editing, Project administration.
