# Supplementary material for: The RNA landscape of Dunaliella salina in response to short-term salt stress
Source: Front Plant Sci. 2023 Dec 4;14:1278954. doi: 10.3389/fpls.2023.1278954 (PMC10726701; doi:10.3389/fpls.2023.1278954)
Supplement: Supplementary file 1 [file DataSheet_1.docx]

Supplementary File 1

The RNA landscape of *Dunaliella salina* in response to short-term salt stress

**Bingbing Zhang*, Caiyun Deng*, Shuo Wang, Qianyi Deng, Yongfan Chu, Ziwei Bai, Axiu Huang**

**Correspondence:** Qinghua He^*^：[demeatry@gmail.com](mailto:demeatry@gmail.com);Qinglian Zhang^*^:qlzhang80@163.com

# Supplementary Figures


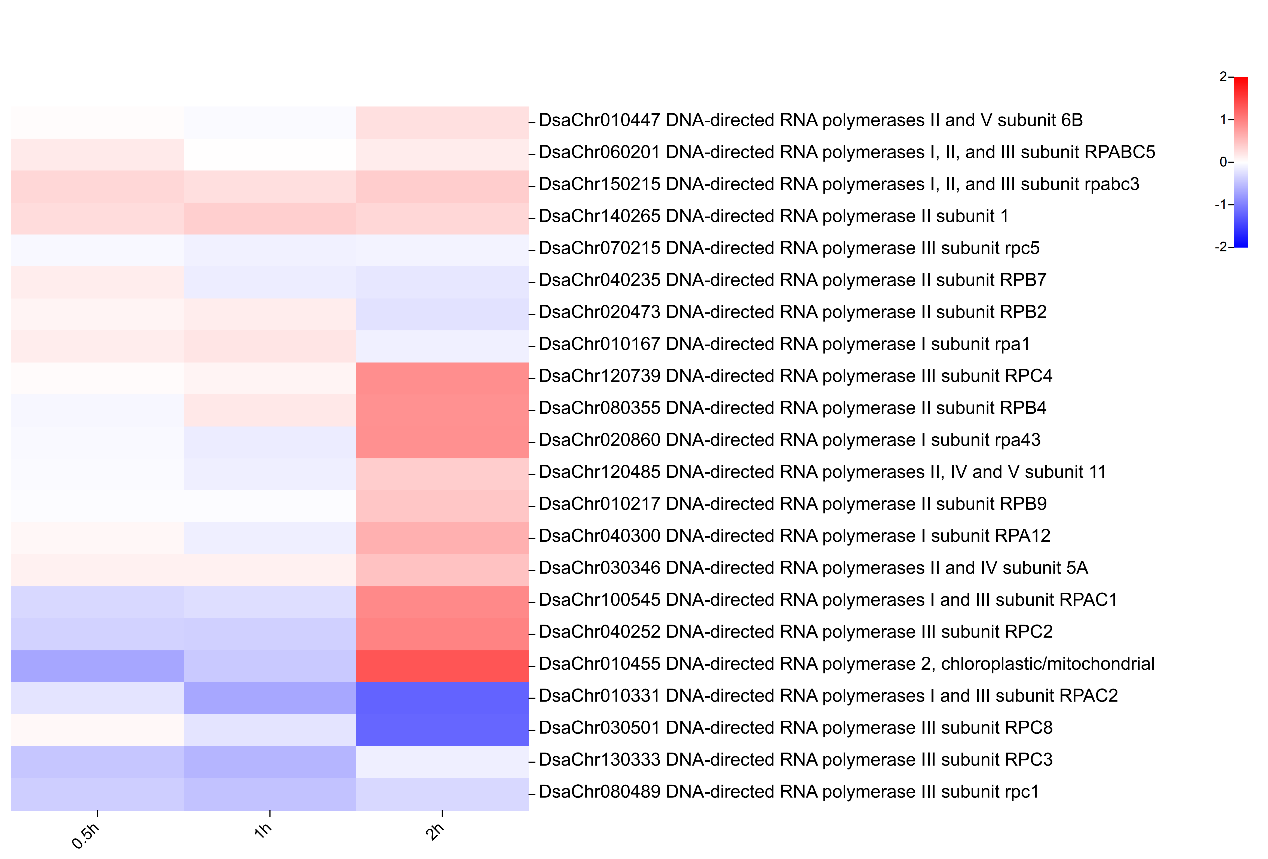
**Fig. S1** Heatmap of DNA-directed RNA polymerases subunits, the colors from blue to red represent the gene expression values from low to high. Values of log2 (Fold change) are used to generate the figure. The following heatmaps are all generated by this method.


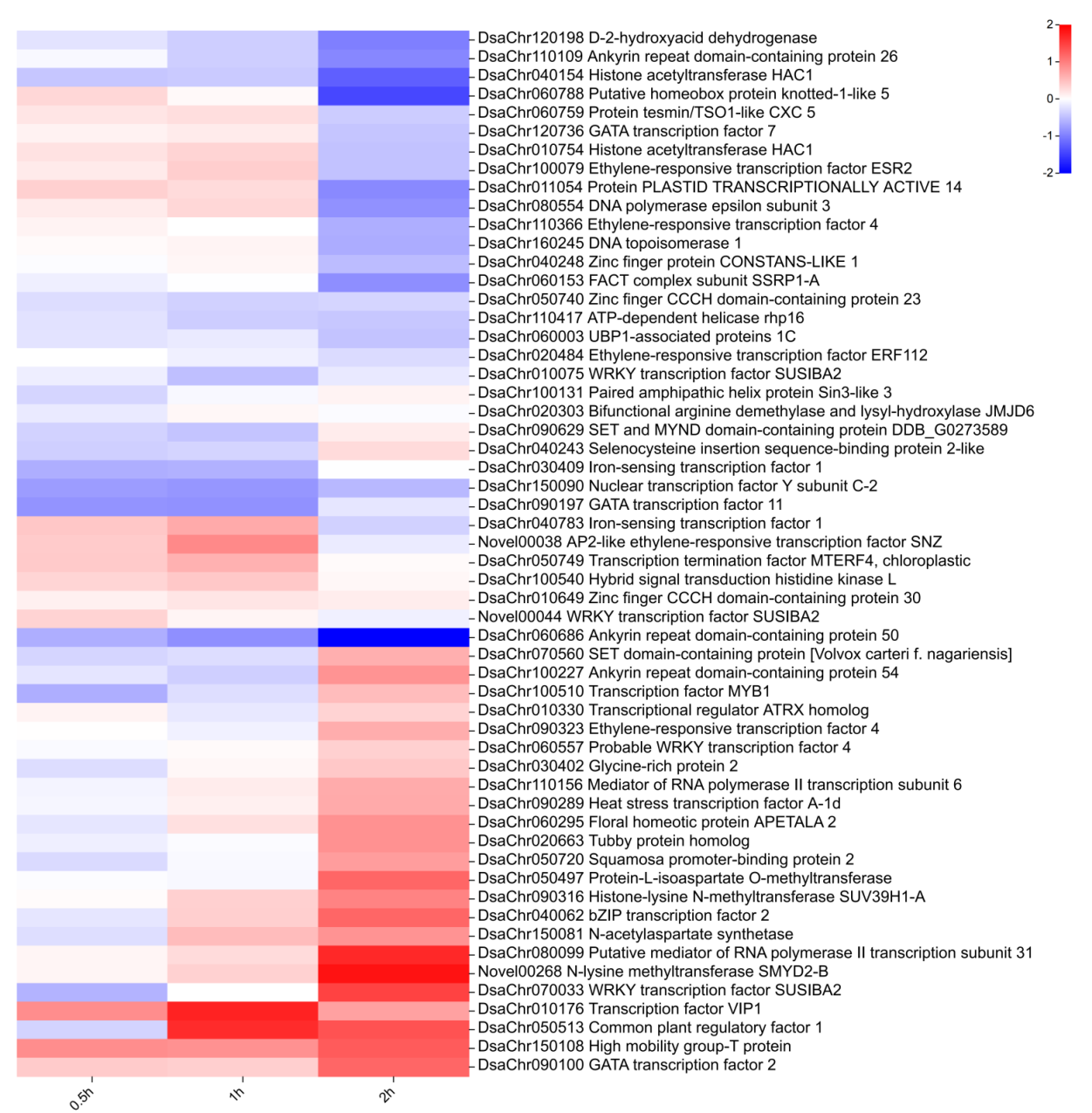
**Fig. S2** Heatmap of transcription factors, the colors from blue to red represent the gene expression values from low to high. Values of log2 (Fold change) are used to generate the figure.


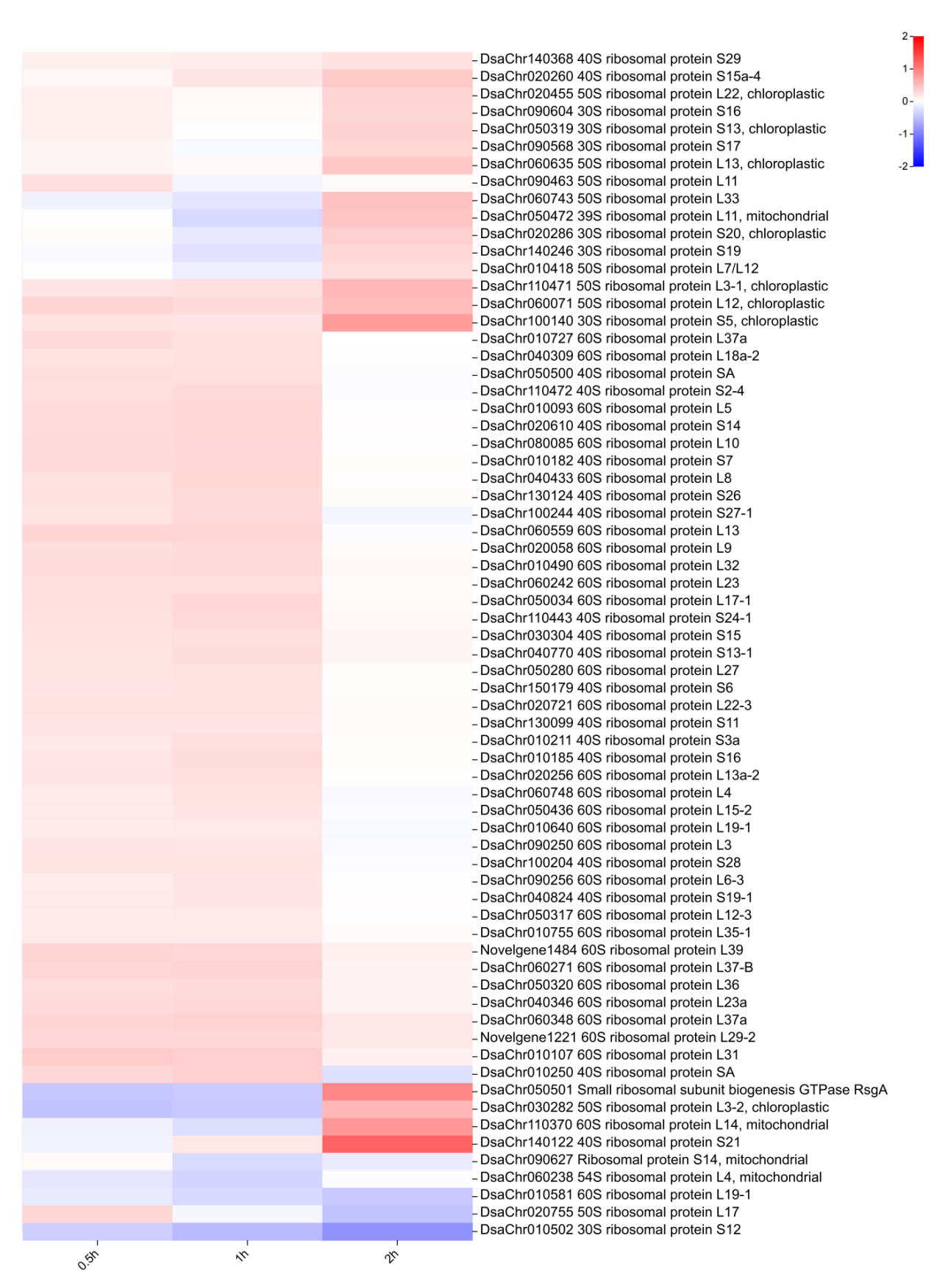
**Fig. S3** Heatmap of ribosomal proteins, the colors from blue to red represent the gene expression values from low to high. Values of log2 (Fold change) are used to generate the figure.


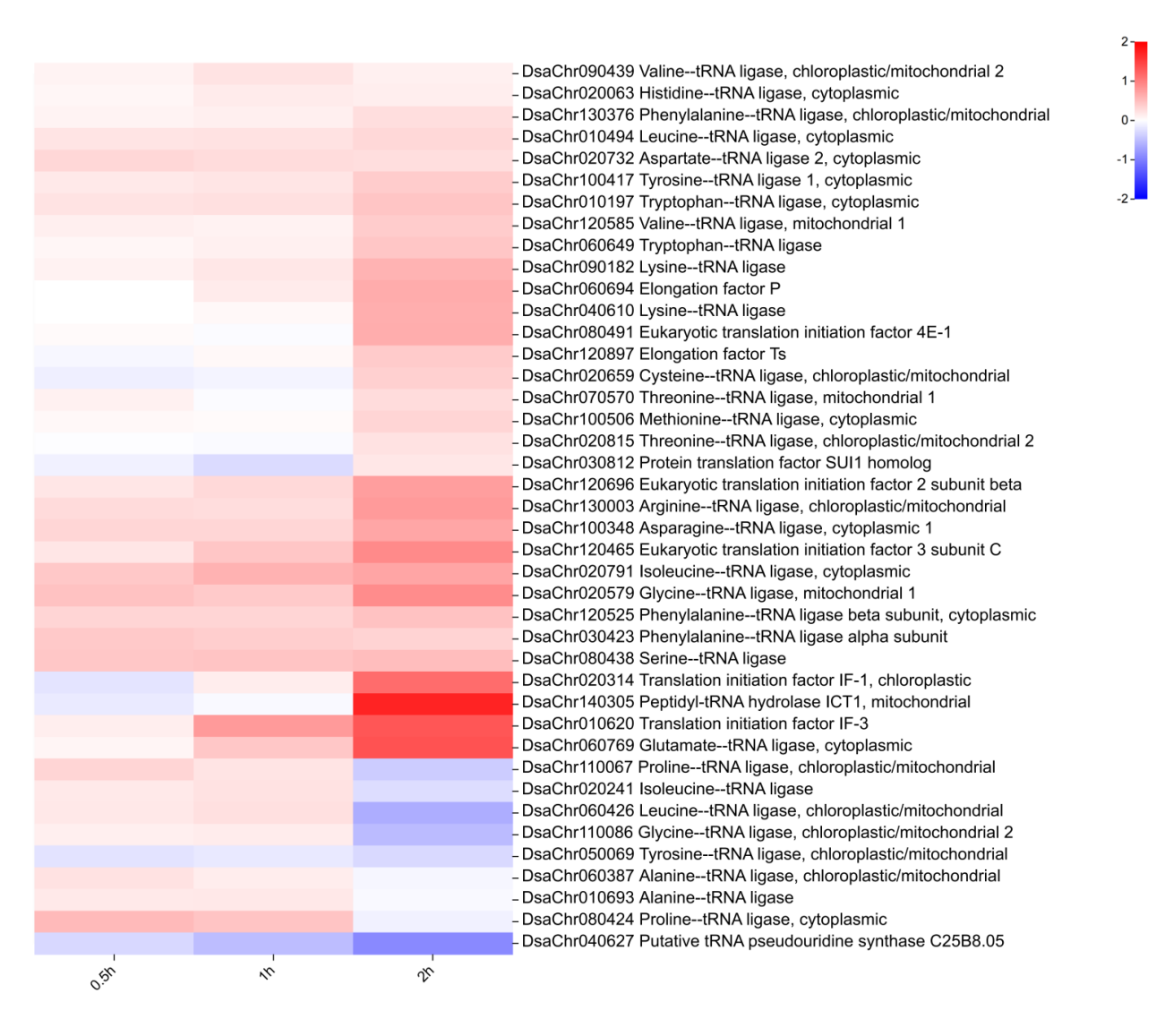
**Fig. S4** Heatmap of tRNA ligases & translational factors, the colors from blue to red represent the gene expression values from low to high. Values of log2 (Fold change) are used to generate the figure.


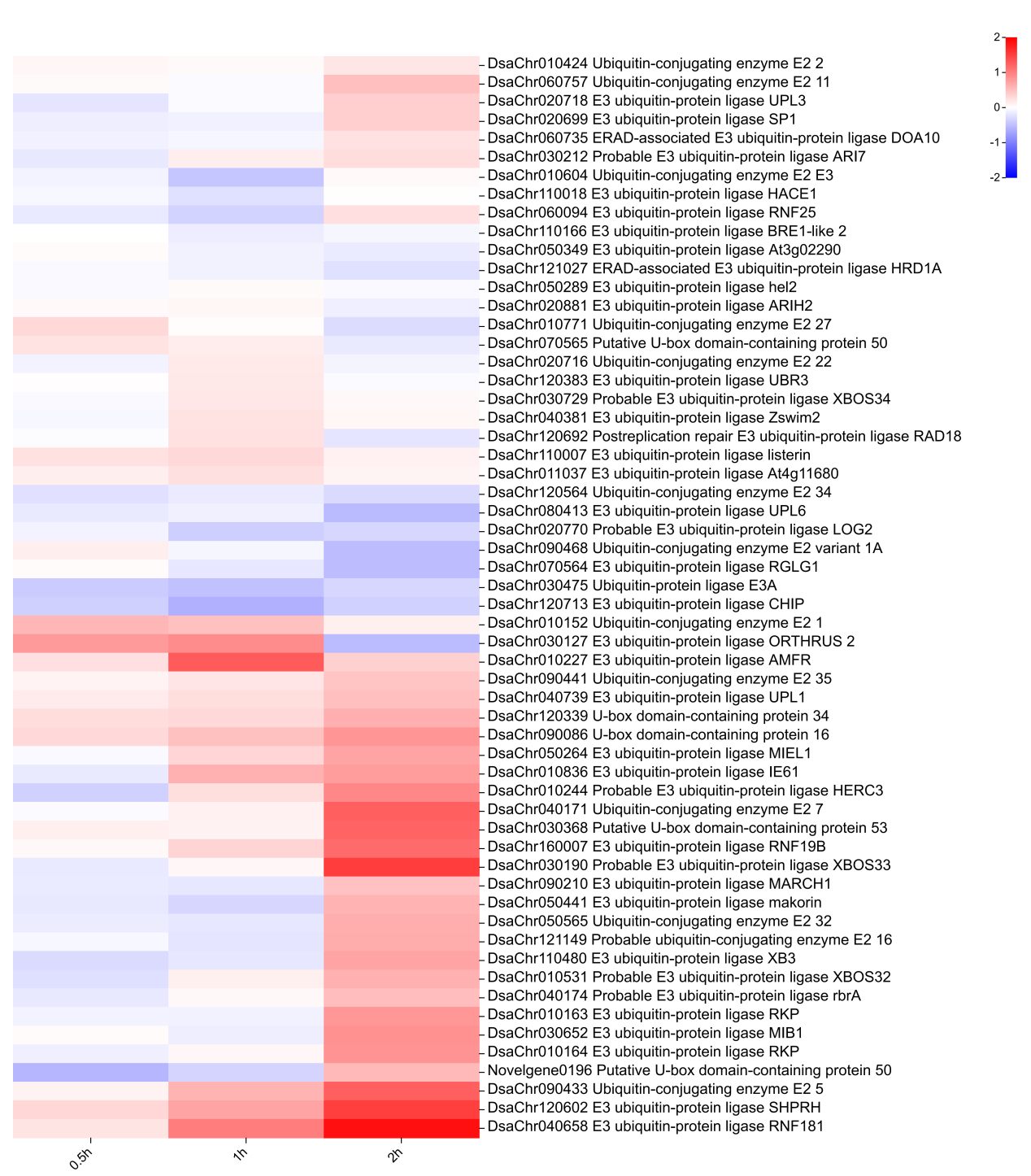


**Fig. S5** Heatmap of ubiquitin-protein ligases & ubiquitin-conjugating enzymes, the colors from blue to red represent the gene expression values from low to high. Values of log2 (Fold change) are used to generate the figure.


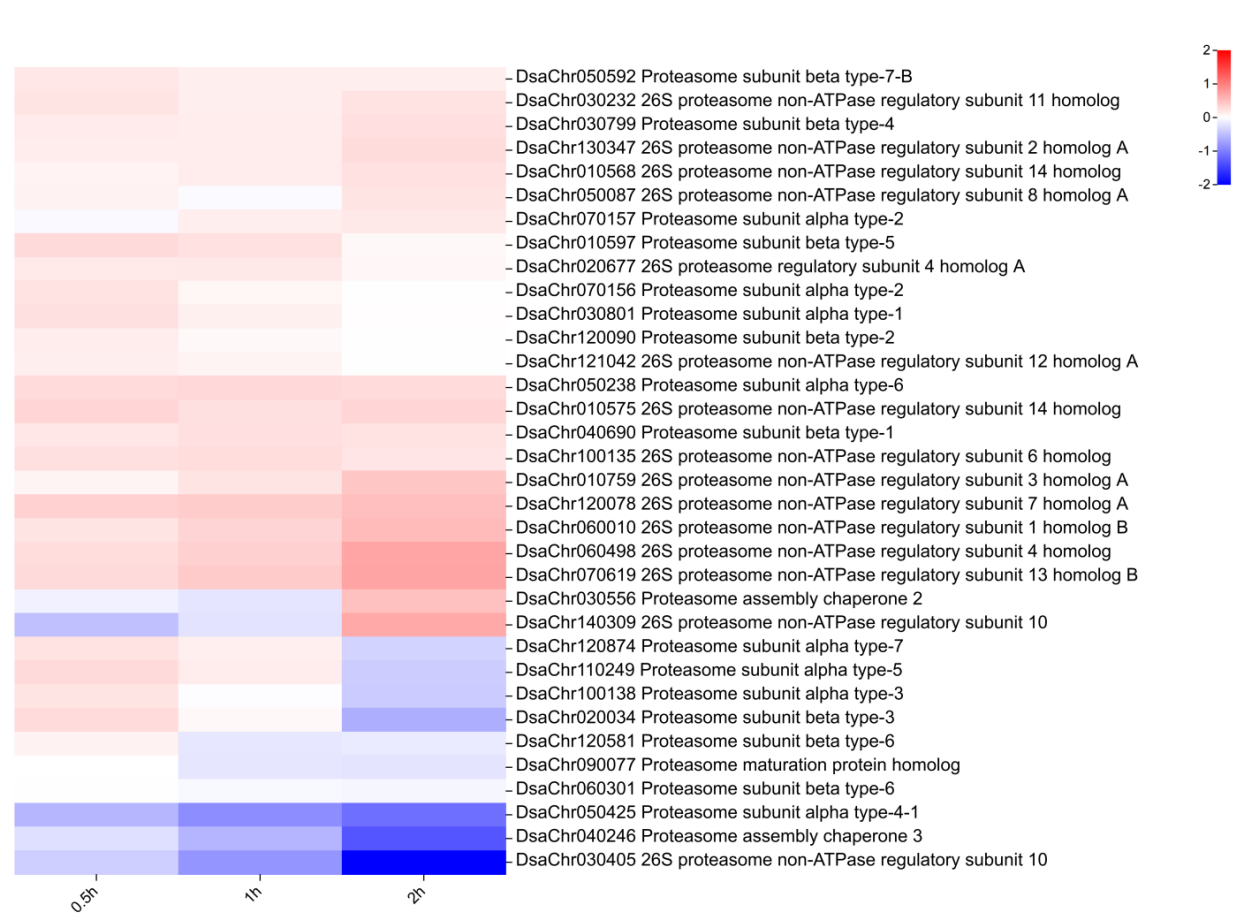


**Fig. S6** Heatmap of proteasome subunits, the colors from blue to red represent the gene expression values from low to high. Values of log2 (Fold change) are used to generate the figure.


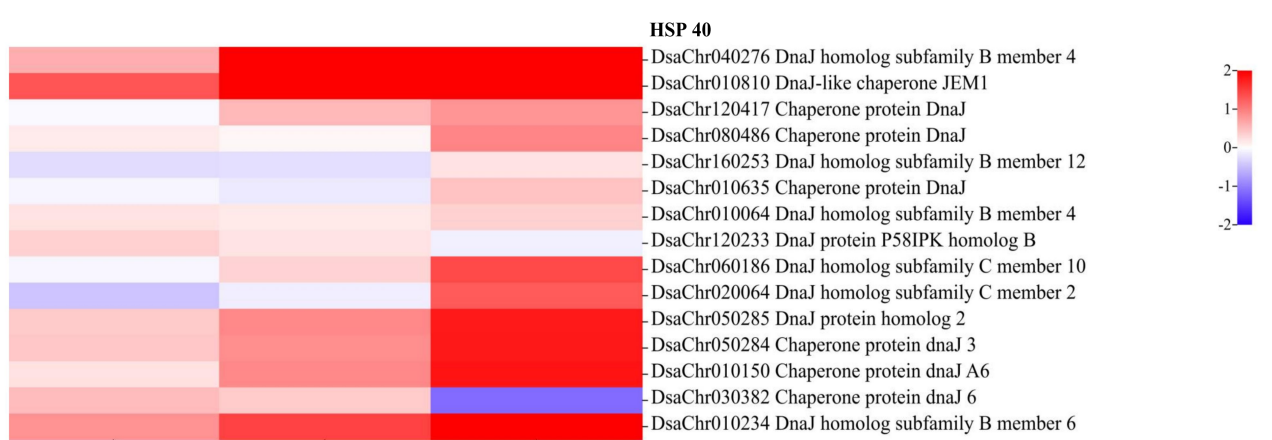


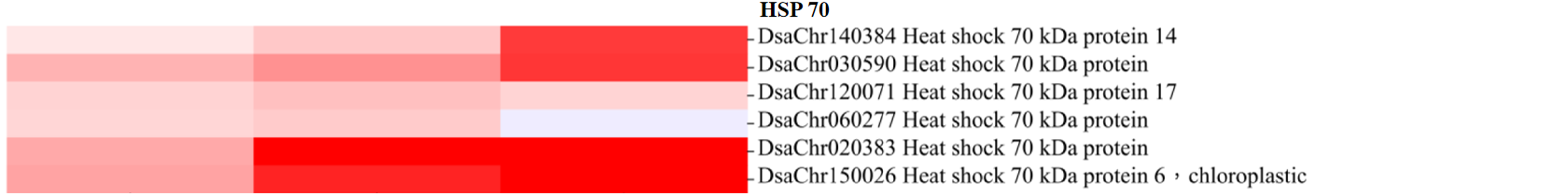


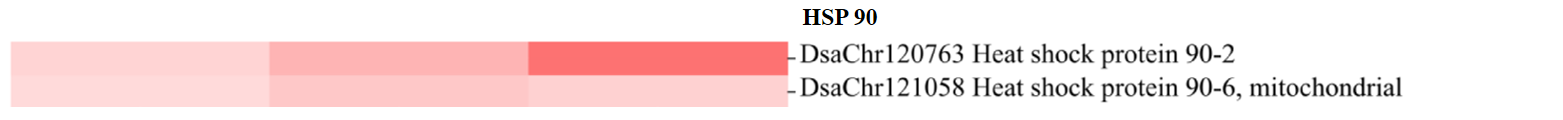


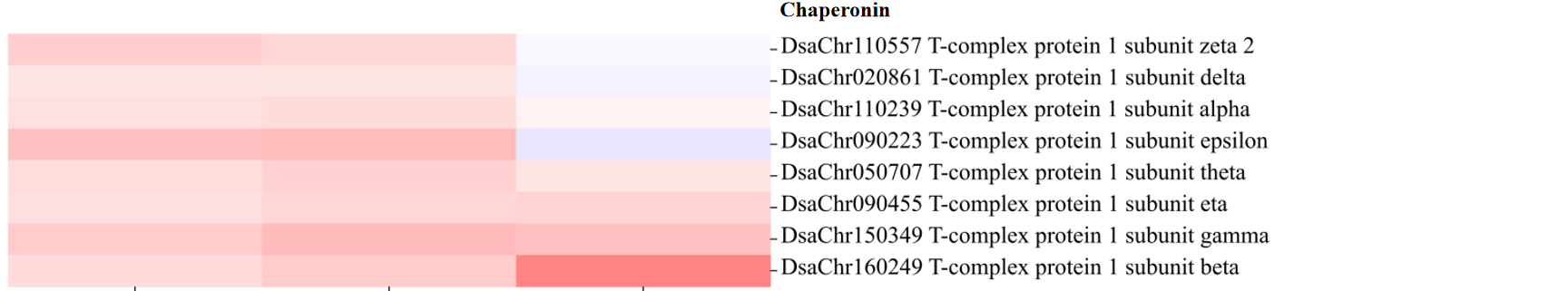


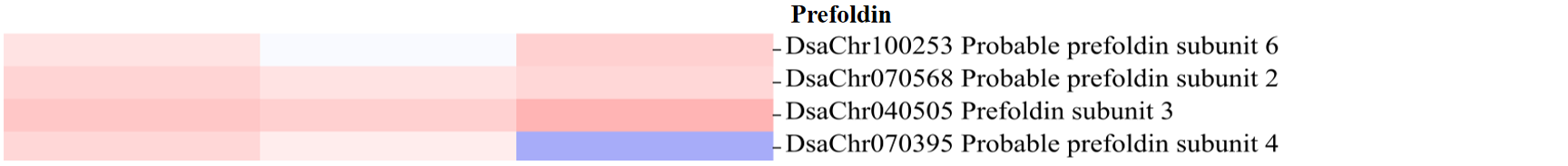


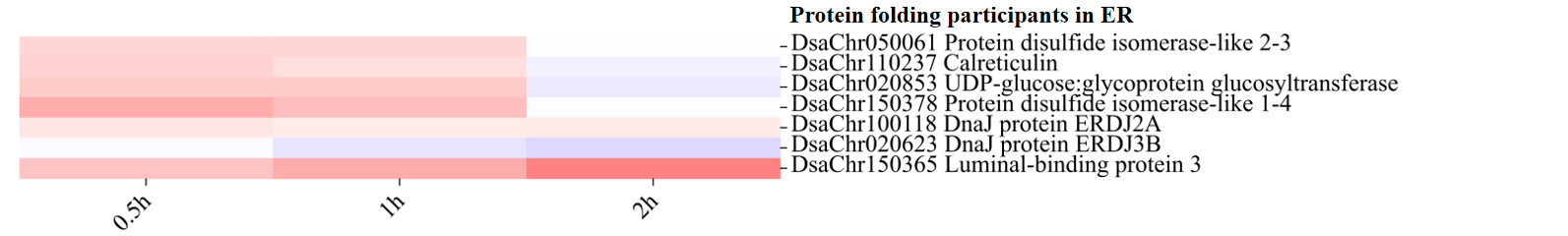


**Fig. S7** Heatmap of protein folding related genes, the colors from blue to red represent the gene expression values from low to high. Values of log2 (Fold change) are used to generate the figure.

Fig S5 Heatmap of ubiquitin-protein ligases & ubiquitin-conjugating enzymes, the colors from blue to red represent the gene expression values from low to high. Values of log_2_ (Fold change) are used to generate the figure.


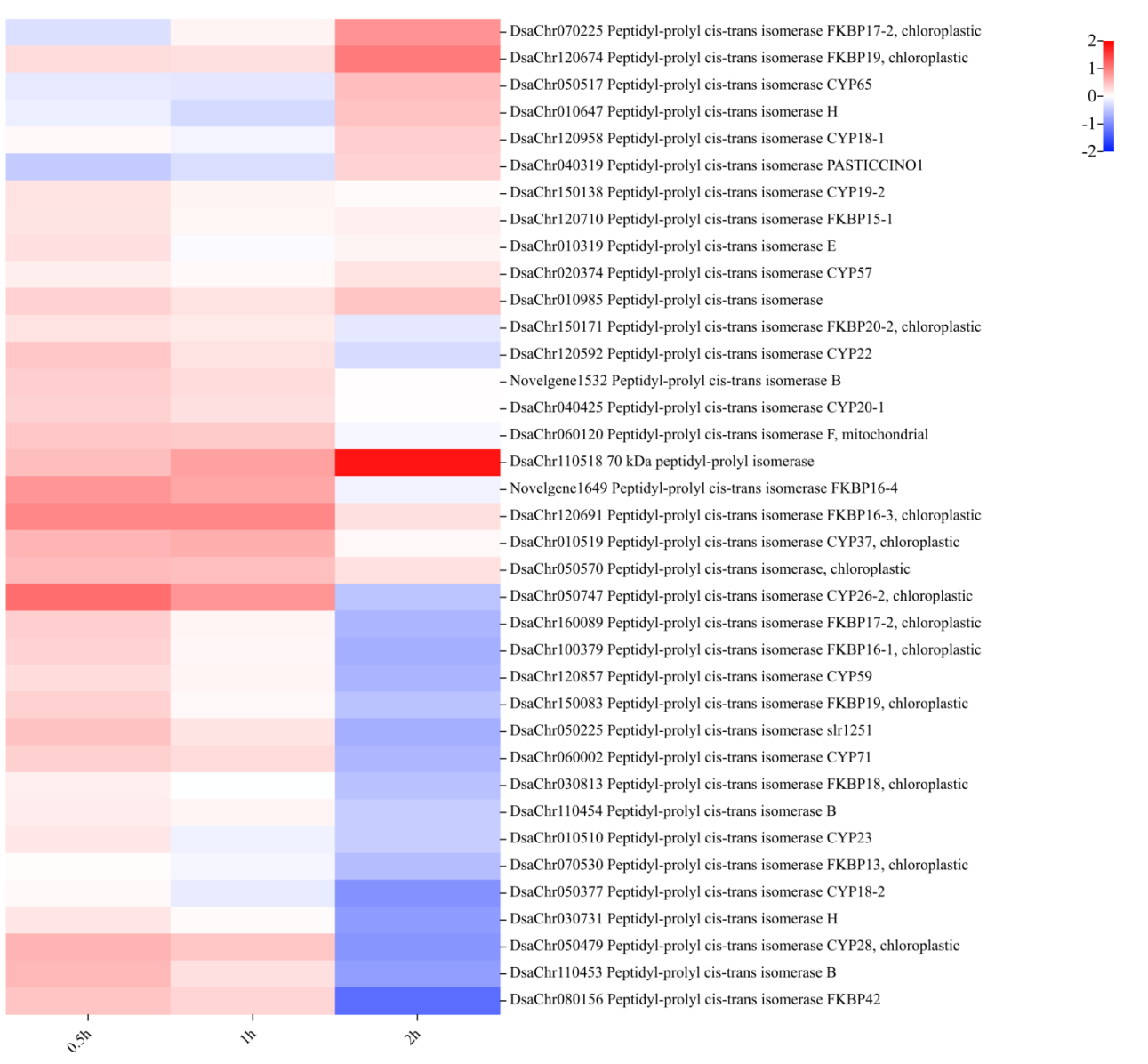
**Fig. S8** Heatmap of peptidyl-prolyl cis-trans isomoerases, the colors from blue to red represent the gene expression values from low to high. Values of log2 (Fold change) are used to generate the figure.


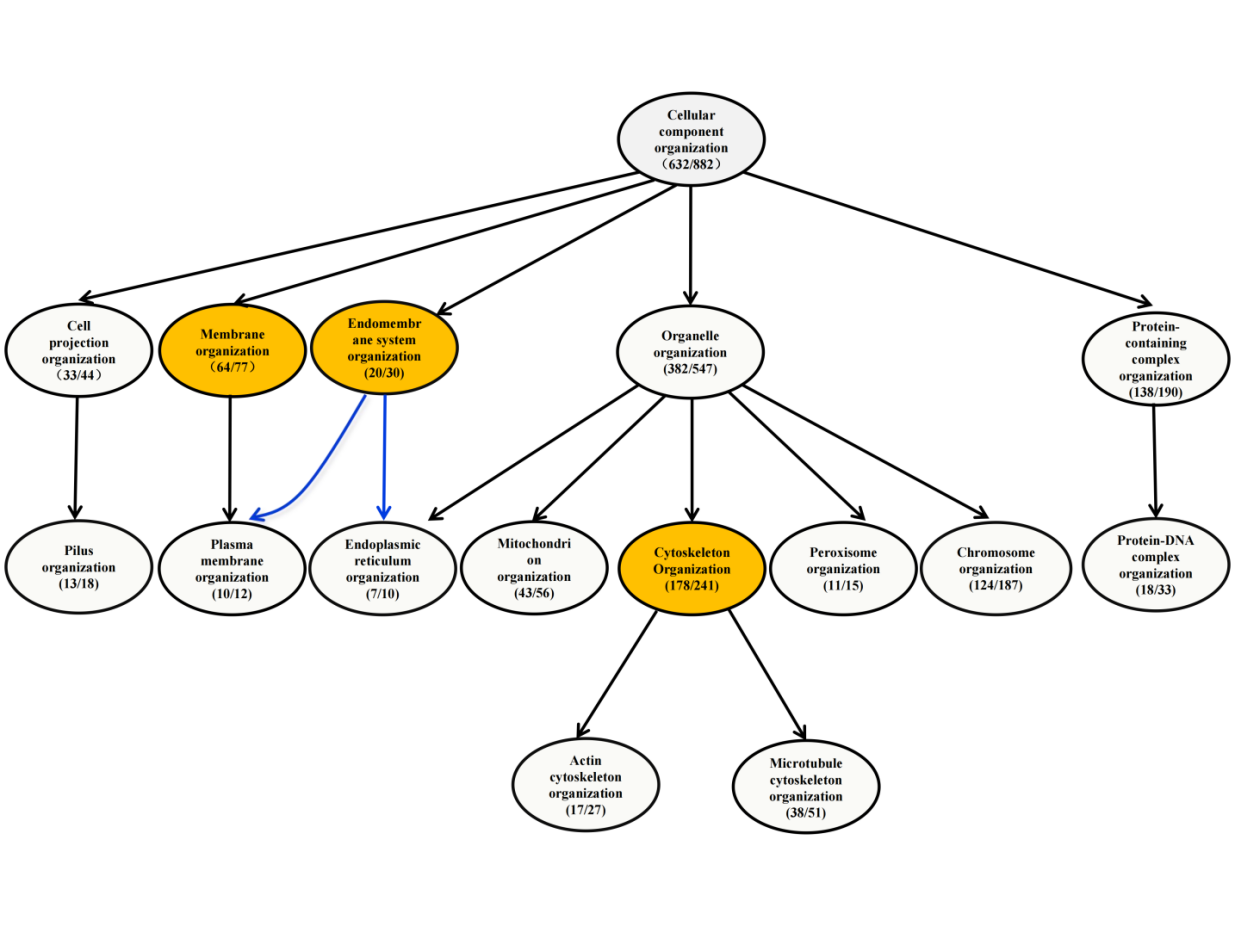


**Fig. S9** Directed acyclic diagram of organization related terms, the ends the arrows point to are the child terms and the other ends are father terms, the three highlighted GO terms are discussed in the paper, the numerators in the brackets are the numbers of the DEGs enriched in the GO terms and the denominators are numbers of the total genes enriched in the GO terms.


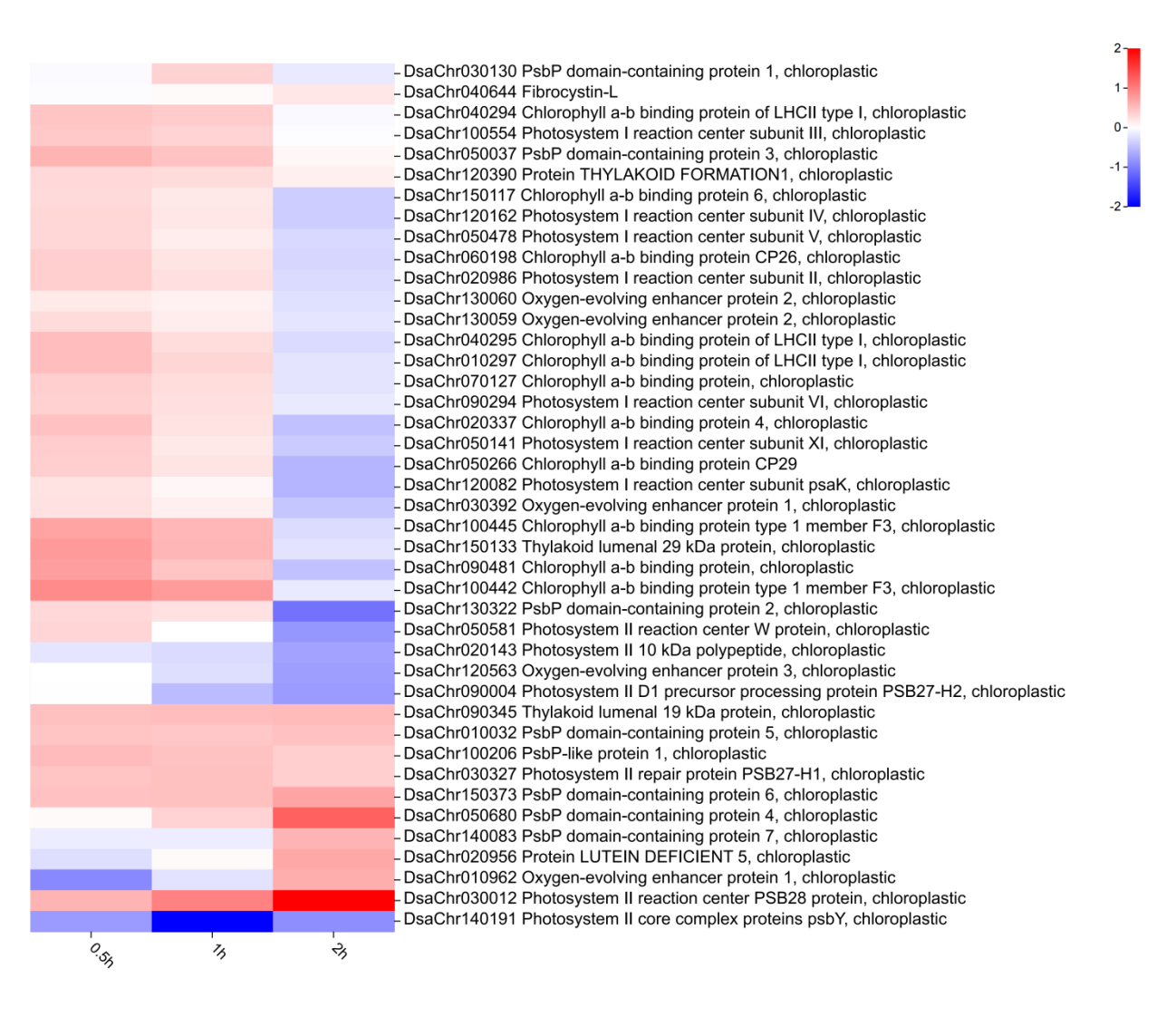


**Fig. S10** Heatmap of photosynthesis related genes, the colors from blue to red represent the gene expression values from low to high. Values of log2 (Fold change) are used to generate the figure.


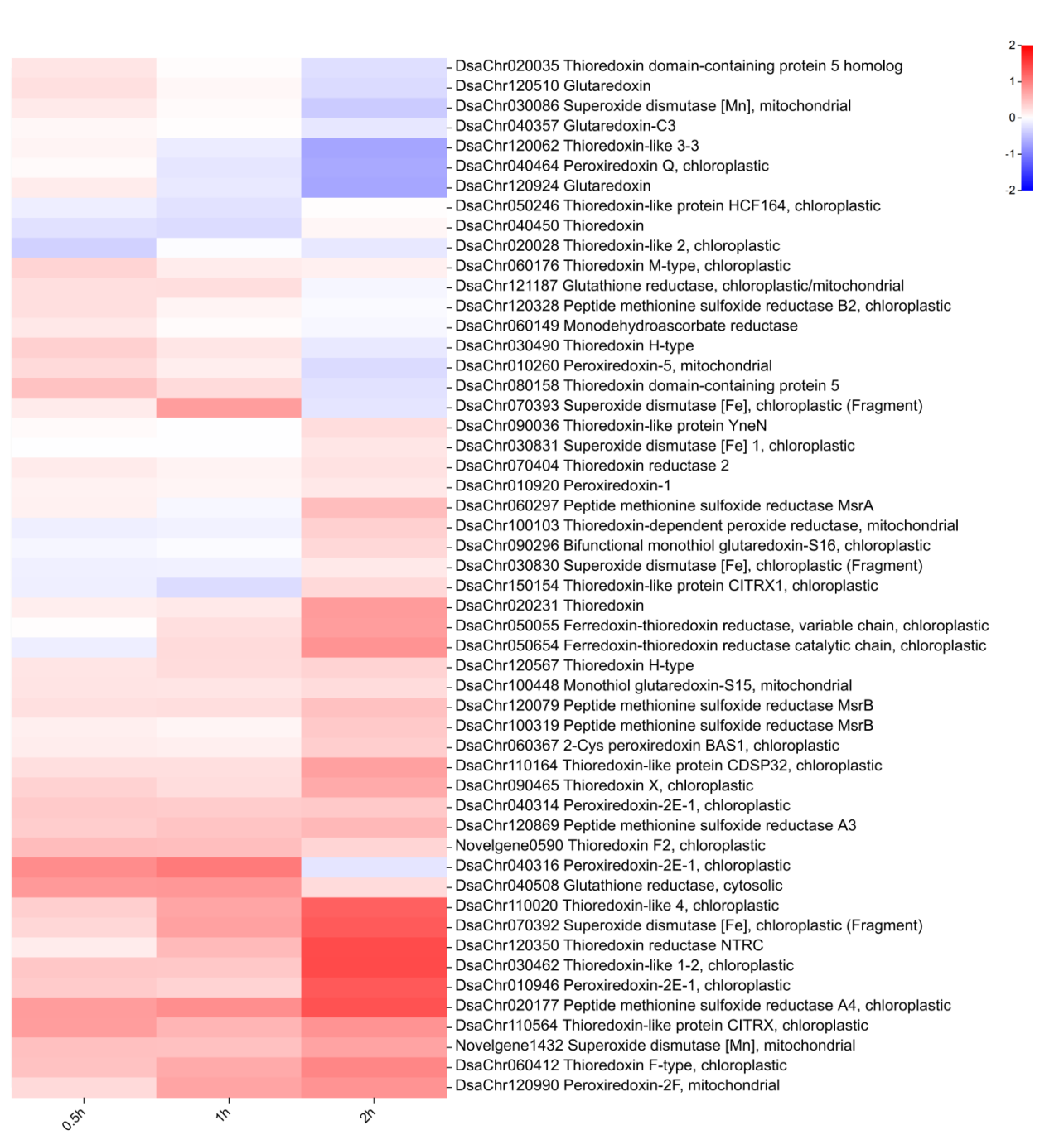


**Fig. S11** Heatmap of cell-redox homeostasis related genes, the colors from blue to red represent the gene expression values from low to high. Values of log2 (Fold change) are used to generate the figure.


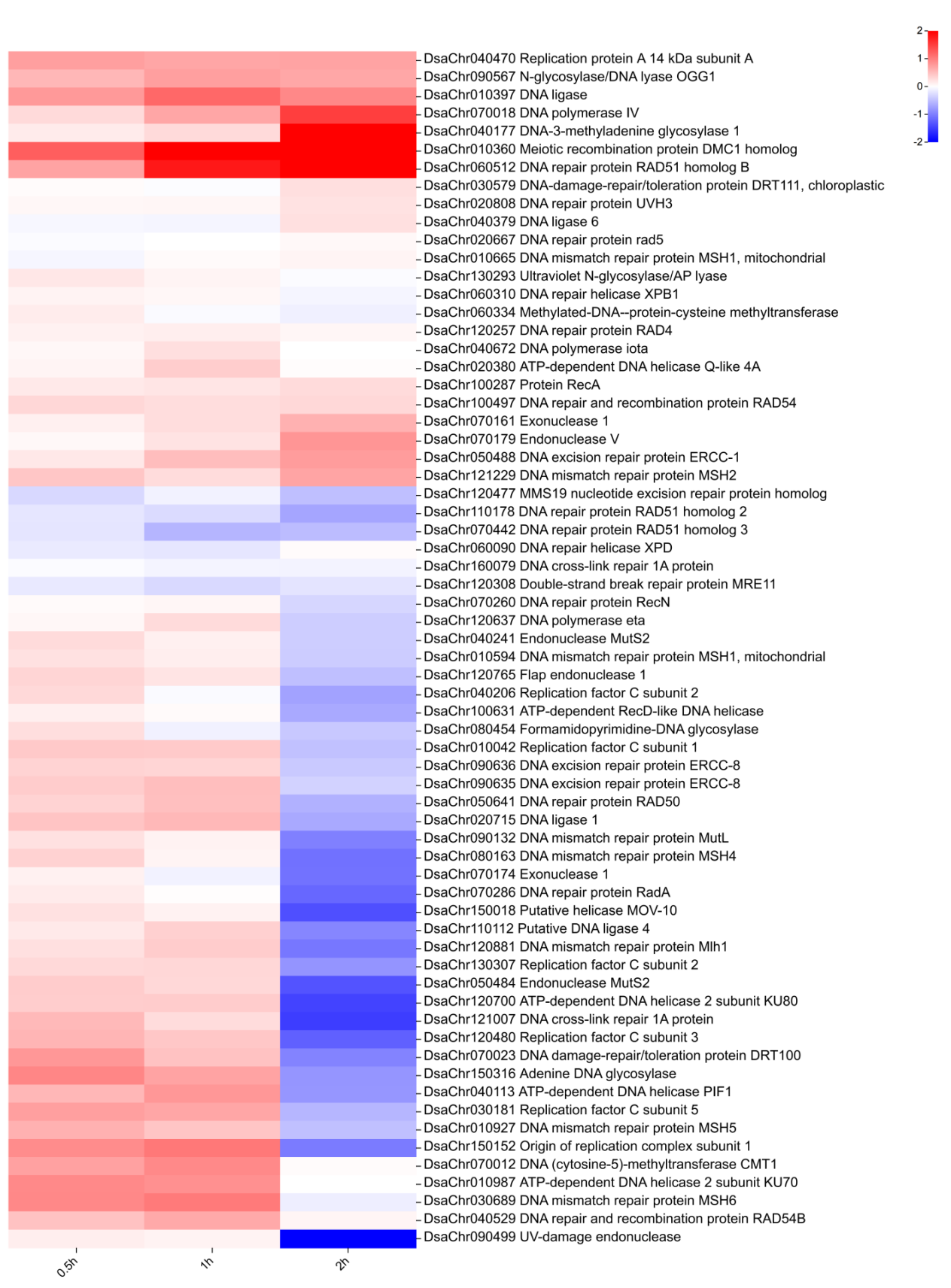


**Fig. S12** Heatmap of DNA-repair related genes,,the colors from blue to red represent the gene expression values from low to high. Values of log2 (Fold change) are used to generate the figure.


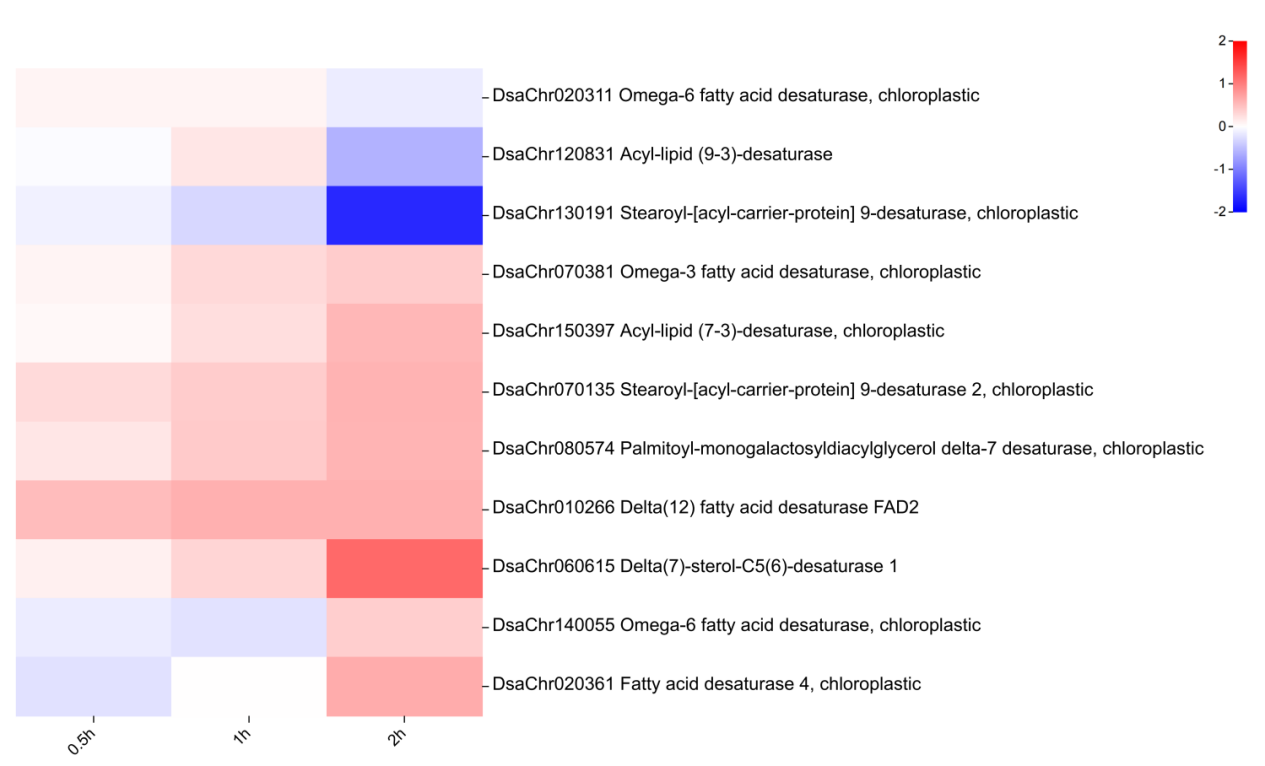


**Fig. S13** Heatmap of fatty acid desaturases, the colors from blue to red represent the gene expression values from low to high. Values of log2 (Fold change) are used to generate the figure.
