# Supplementary material for: The RNA landscape of Dunaliella salina in response to short-term salt stress
Source: Front Plant Sci. 2023 Dec 4;14:1278954. doi: 10.3389/fpls.2023.1278954 (PMC10726701; doi:10.3389/fpls.2023.1278954)
Supplement: Supplementary file 2 [file DataSheet_2.docx]

Supplementary File 2

The RNA landscape of *Dunaliella salina* in response to short-term salt stress

**Bingbing Zhang*, Caiyun Deng*, Shuo Wang, Qianyi Deng, Yongfan Chu, Ziwei Bai, Axiu Huang**

*** Correspondence:** Qinglian Zhang^*^:qlzhang80@163.com;Qinghua He^*^：[demeatry@gmail.com](mailto:demeatry@gmail.com)

# Supplementary Figures

**Real-time PCR confirmation of selected key genes in the biological processes**

The significance values determined by one-way ANOVA are indicated by asterisks in the graphs (^*^ *P*<0.05).^**^ *P*< 0.01, ^***^ *P*< 0.001,^****^ *P*< 0.0001)

Genes involved in transcription


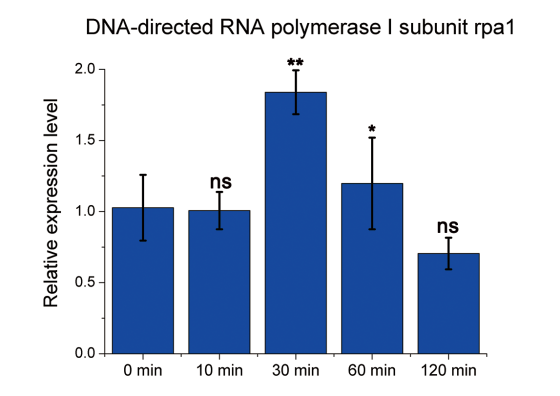

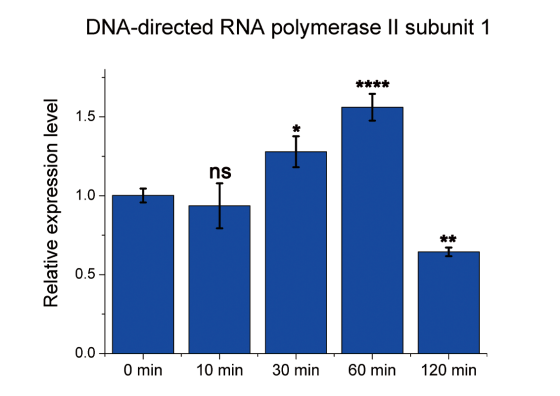


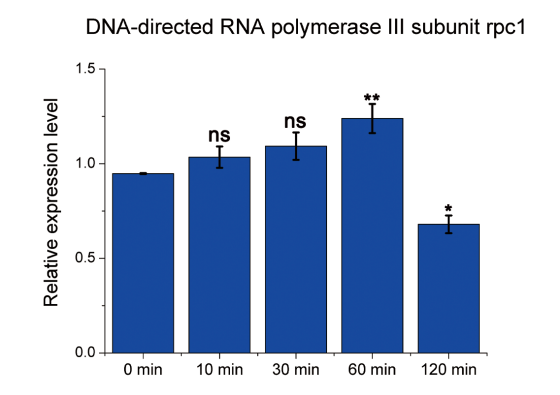

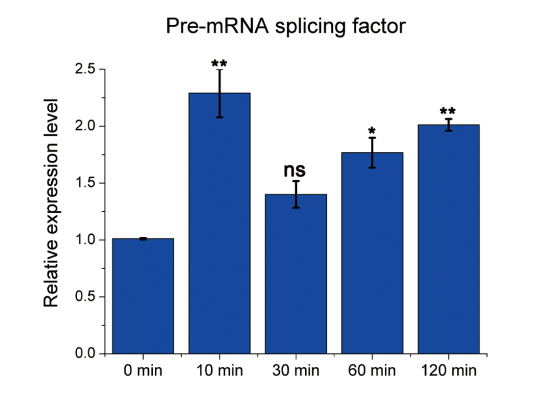


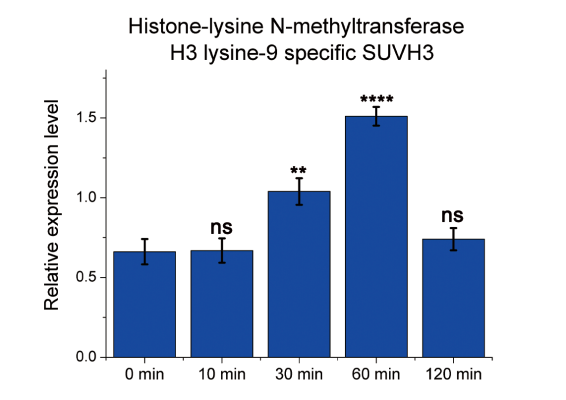


Genes involved in protein synthesis


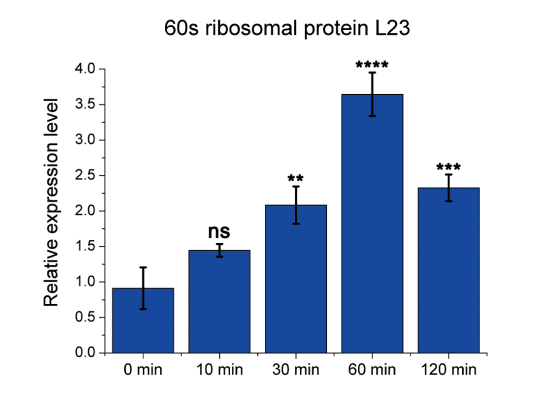

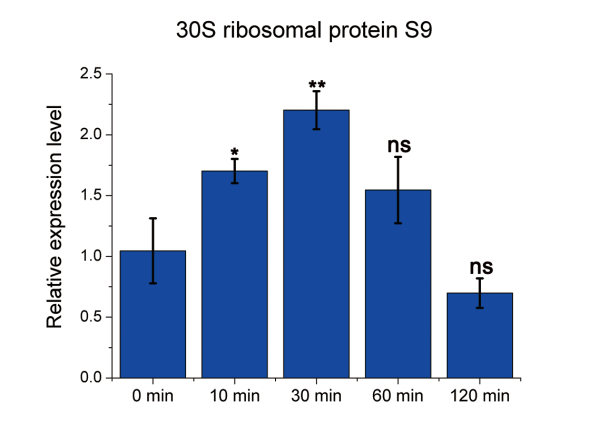


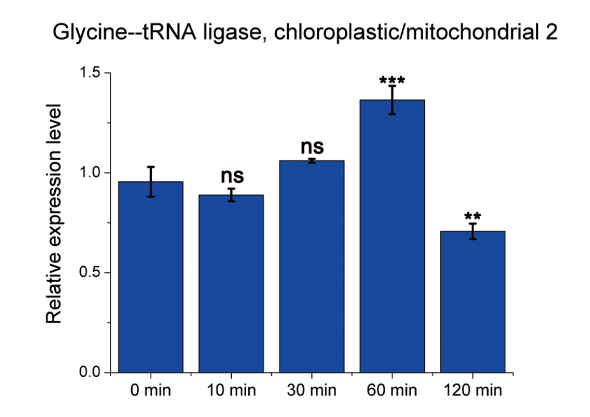

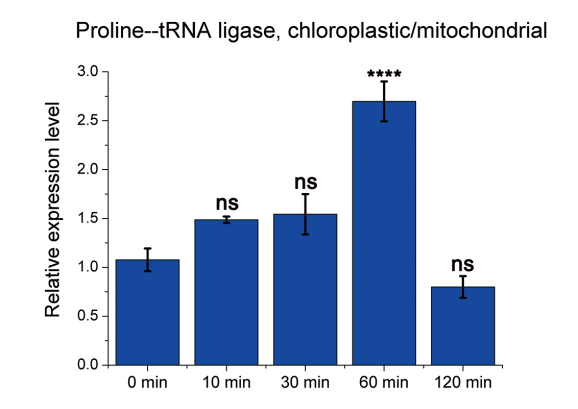


Genes involved in protein degradation


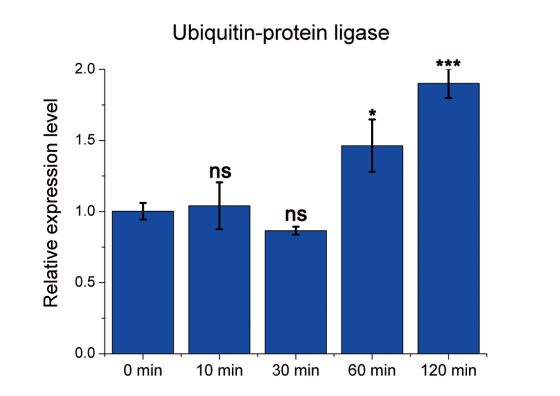

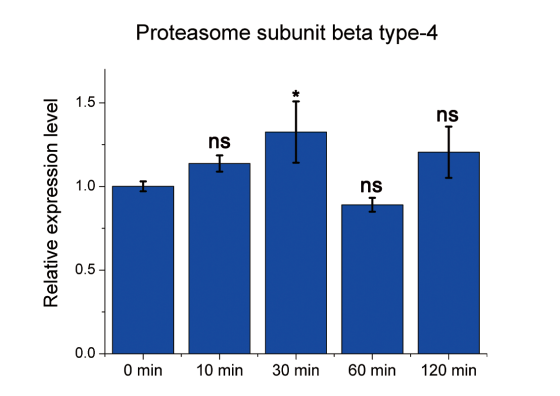


Gene involved in protein folding


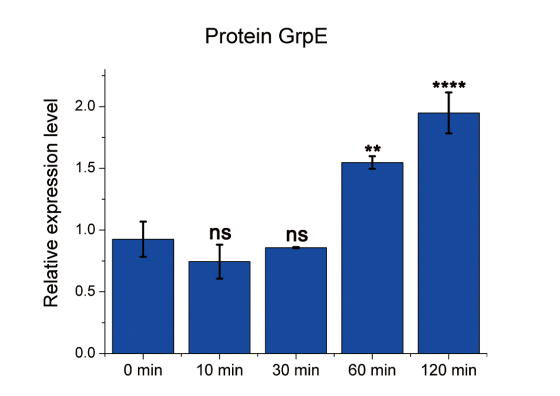


Gene involved in protein modification


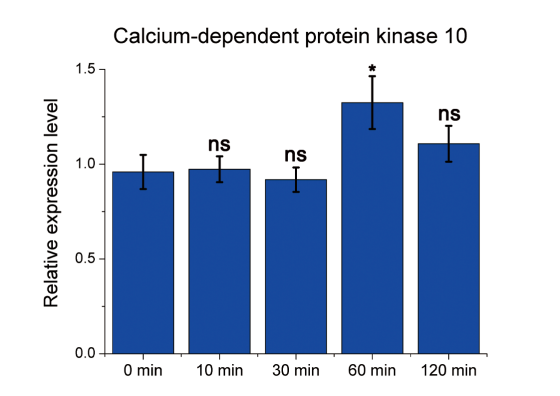


Genes involved in protein transportation


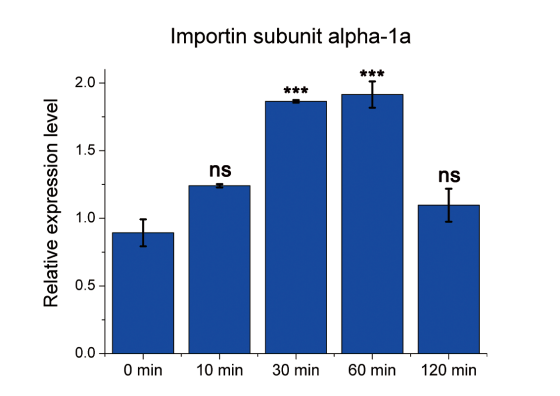

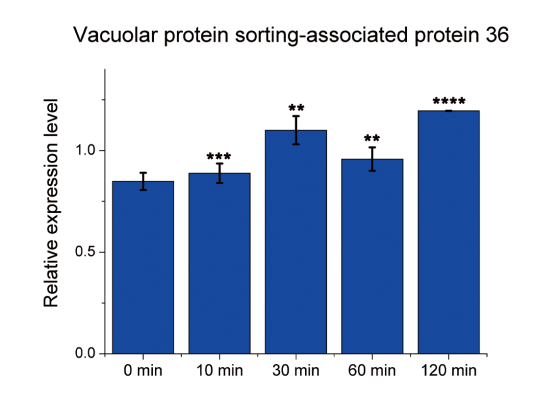


Genes involved in cellular component organization


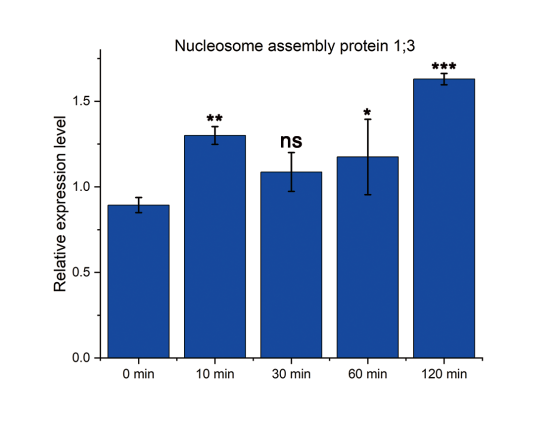


Genes involved in DNA repair


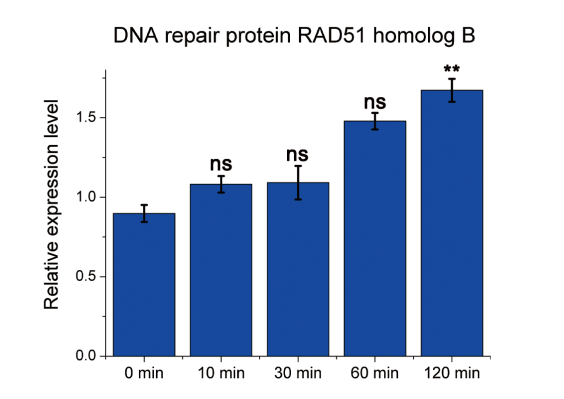

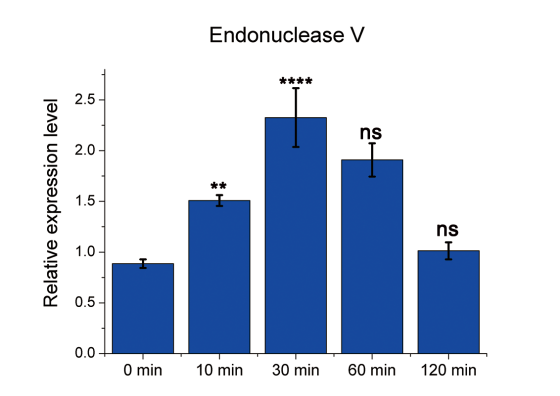


Genes involved in cell redox homeostasis


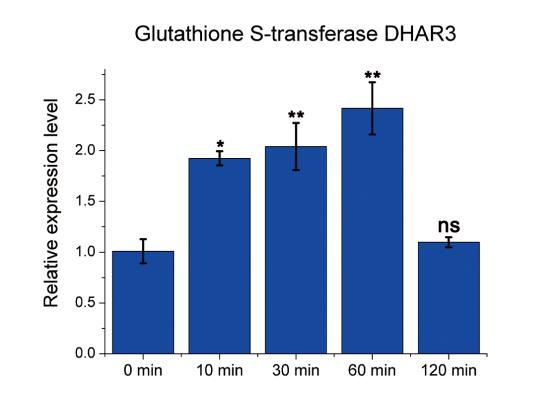


Genes involved in ion homeostasis


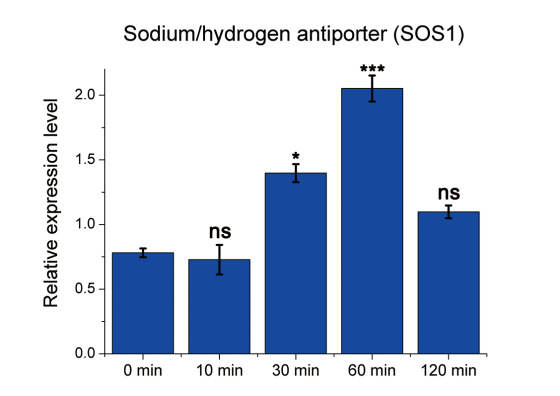

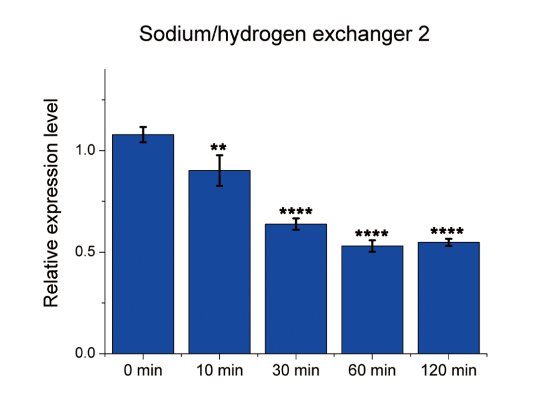


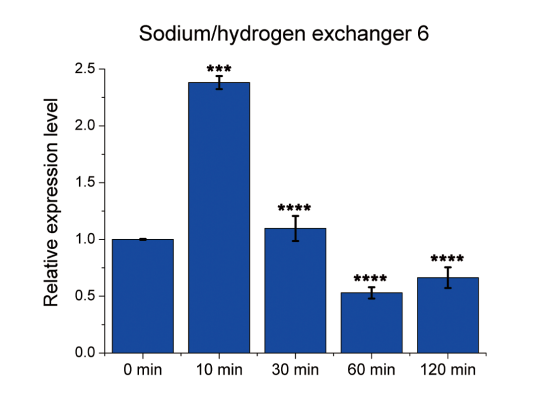

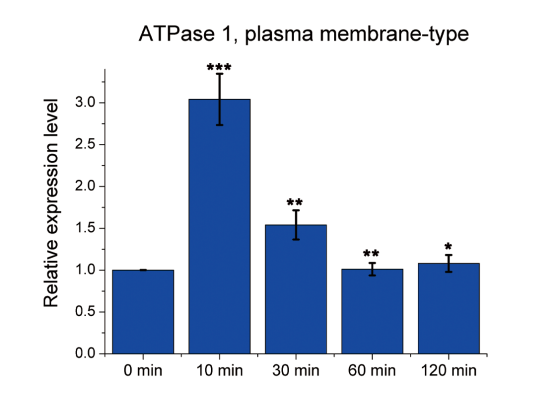


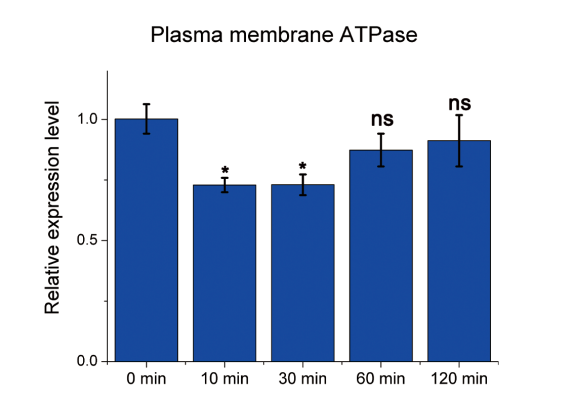

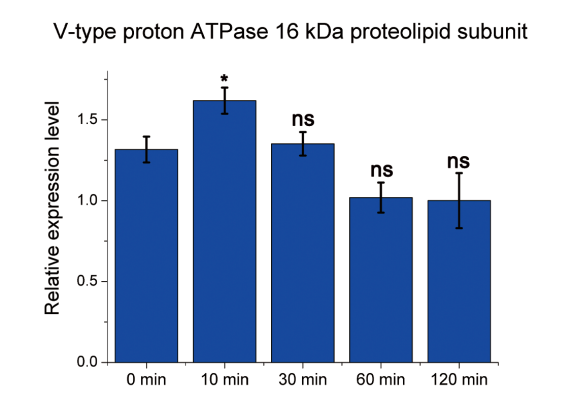


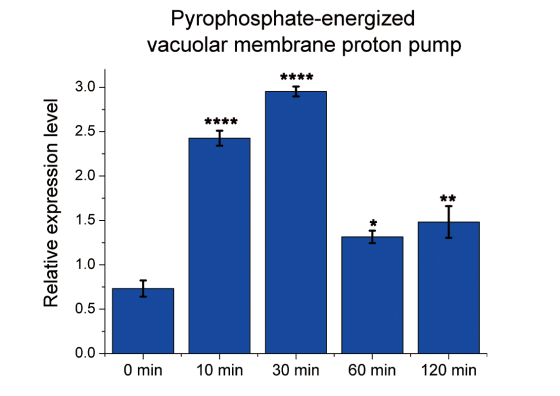





Genes involved in pentose phosphate pathway


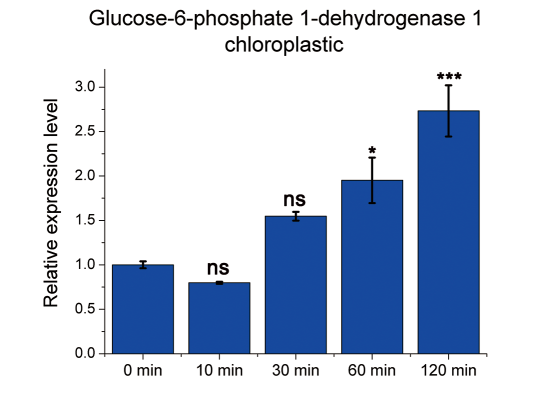

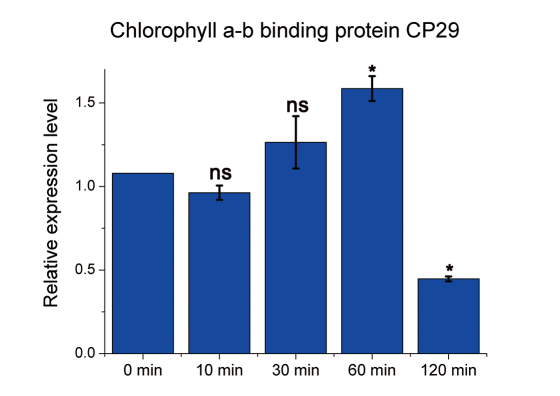


Genes involved in photosynthesis


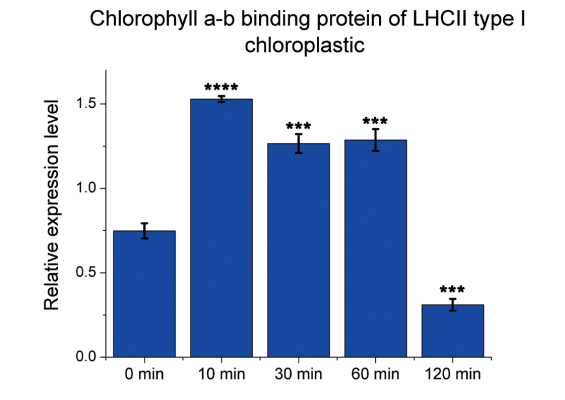


Genes involved in fatty acids biosynthesis


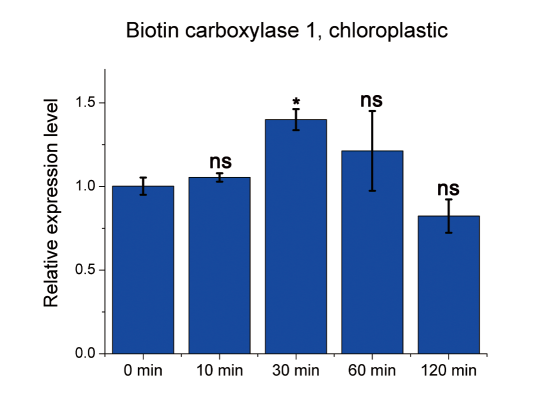

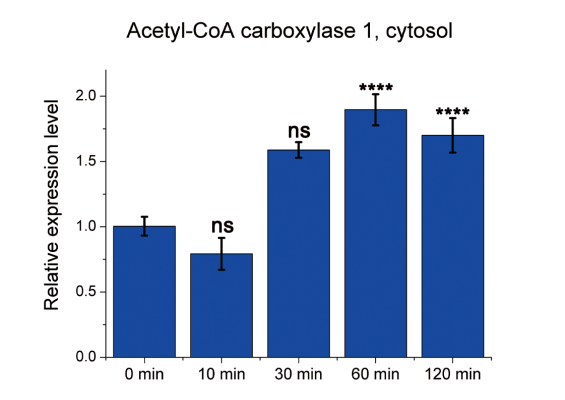


Genes involved in TAG biosynthesis


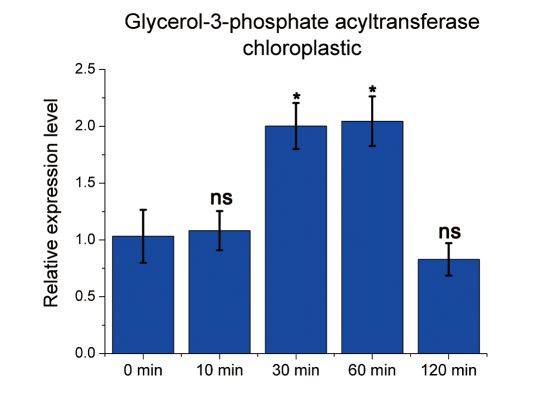


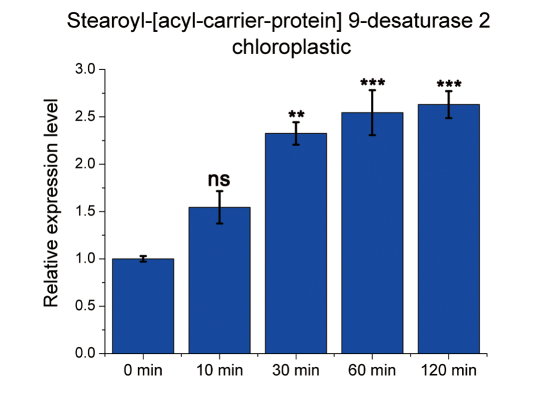

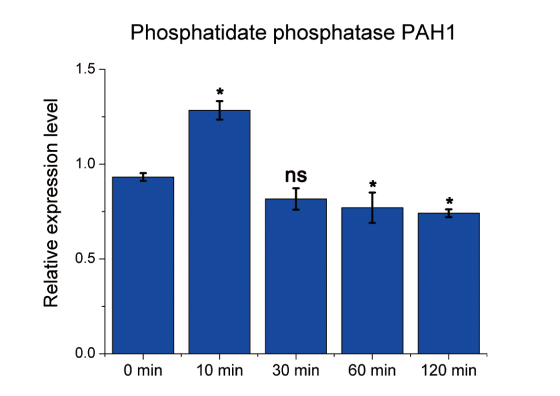


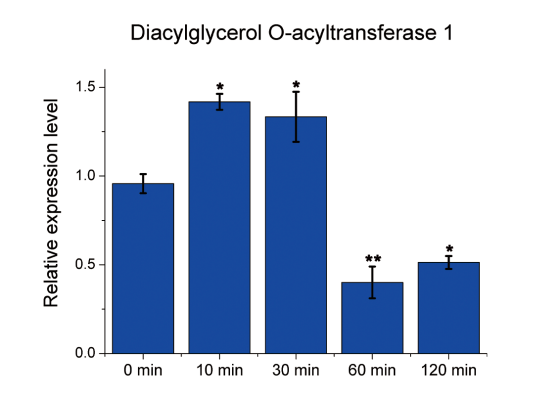

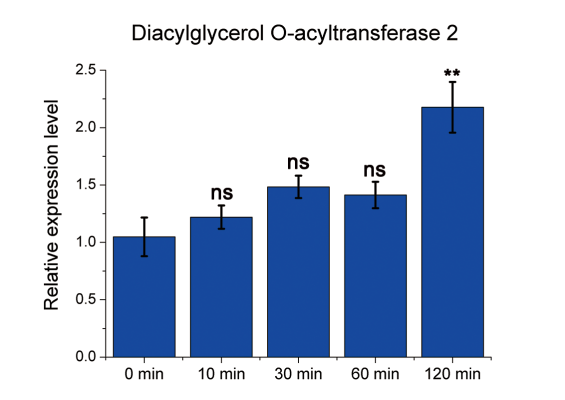


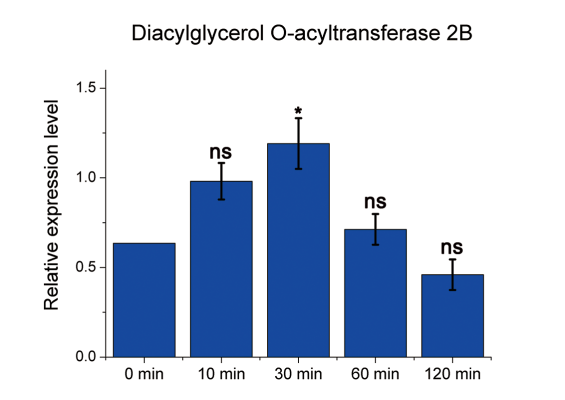

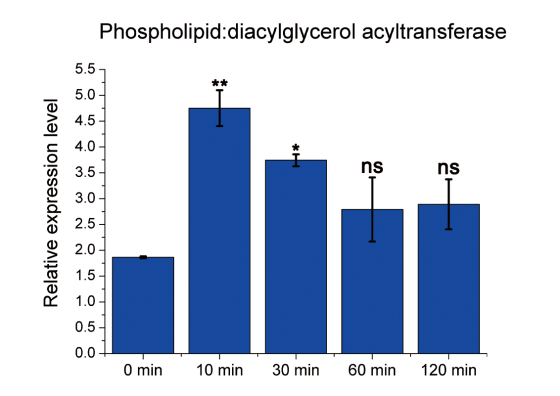


Genes involved in glycerophospholipid metabolism


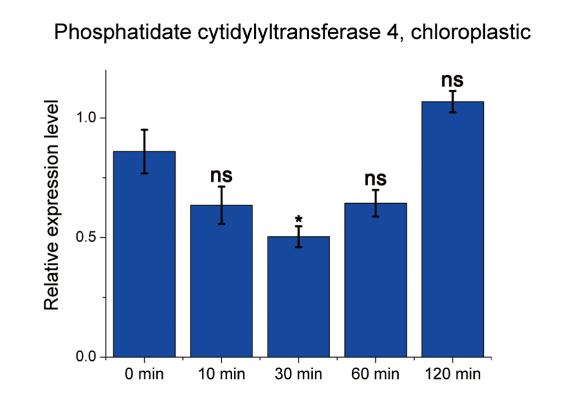

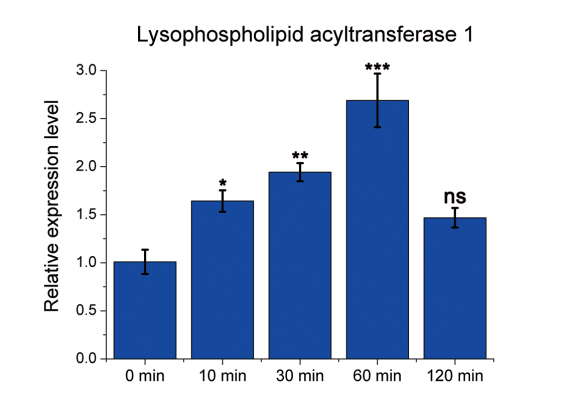


# Supplementary Table

Sequences of the primers used in real-time PCR

| Gene id | Gene name | Primer sequences |
| --- | --- | --- |
| DsaChr010167 | DNA-directed RNA polymerase I subunit rpa1 | Ps CATCATGCTGACCAACCGGC  Pa AAGTCGGCGTTGAAGGTGGA |
| DsaChr140265 | DNA-directed RNA polymerase II subunit 1 | Ps CACTCGCCATGGCATCAACC  Pa TGCAGTTGTCACGCTCTCCA |
| DsaChr080489 | DNA-directed RNA polymerase III subunit rpc1 | Ps GATGTCTGCACGGCACATGG  Pa GATCCATGTCGCAGCCTTGC |
| DsaChr090067 | Pre-mRNA splicing factor | Ps CCTTACCAGCTCTGCGGACA  Pa ACCACCTCTGCTTGGTGGTC |
| DsaChr090217 | Histone-lysine N-methyltransferase, H3 lysine-9 specific SUVH3 | Ps GGTCGAGTTCCGCTCCATGA  Pa ACCGGCACCTTGCTCTTCTT |
| DsaChr040346.1 | 60s ribosomal protein L23 | Ps GTGGTCTTCCACCGACCCAA  Pa ATCTTCTTGTTGGCGCGCAG |
| DsaChr070299 | 30S ribosomal protein S9 | Ps CTGCAAGACAACAGCACGCA  Pa GTCCTTGCGGTACACGGAGT |
| DsaChr110086 | Glycine--tRNA ligase, chloroplastic/mitochondrial 2 | Ps AACCTGCCCAAGGAAGTGCT  Pa CGTTGGCCACGGTGATGAAG |
| DsaChr110067 | Proline--tRNA ligase, chloroplastic/mitochondrial | Ps ACAGATGGGAGATGCGGACG  Pa AGCTGCTTCCTCCACAGCAA |
| DsaChr040171 | Ubiquitin-protein ligase | Ps CCATCCTGCACAACCCTGGA  Pa GAACAGCTCTCGGTCCTCCC |
| DsaChr030799 | Proteasome subunit beta type-4 | Ps TACCGCGACAAGCAGTCCAT  Pa CCCTGATGGGCTGAGGAAGG |
| DsaChr040344 | Protein GrpE | Ps GGTTCGGCATTGAGGCAGTG  Pa CTGGGAGATGTGGCCCTCAG |
| DsaChr060187 | Calcium-dependent protein kinase 10 | Ps ACAGCCAGGACCTGCACAAT  Pa CATGGCCGCAATGAACTGCT |
| DsaChr040396 | Importin subunit alpha-1a | Ps GCCATCAGCAATGCCACCTC  Pa CTCACACAATGGCCGGATGC |
| DsaChr130018 | Vacuolar protein sorting-associated protein 36 | Ps GCCTGTTCAATCGGGCAAGG  Pa GGCACTCTGGATGGCTGTGA |
| DsaChr120053 | Nucleosome assembly protein 1;3 | Ps AGGAGTTCGTGGAGTCGCTG  Pa CGTCTCCACCTCGTCATGCT |
| DsaChr060512 | DNA repair protein RAD51 homolog B | Ps GCAGCCCATCCTCTCGACAT  Pa ATGTGCTACCGCCTCCACAG |
| DsaChr070179 | Endonuclease V | Ps GACCGATCGGCCATTGAGGA  Pa GGCTGCTGCCACAATCCTTC |
| DsaChr010682 | Glutathione S-transferase DHAR3 | Ps CGCACAGCGCTCTCAATCAG  Pa CTCTGGGTCGCCTTTCACCA |
| DsaChr110059 | Sodium/hydrogen antiporter (SOS1) | Ps GCGCTGAGCACCTTTCTGTC  Pa ACTGCTACTGCATCCGTGCT |
| DsaChr121177 | Na+/H+ exchanger 2 of Arabidopsis thaliana | Ps GGCCAAGCAGGATCGTTGTG  Pa TGTGTGGTGGACAGCAGGTT |
| DsaChr160120 | Sodium/hydrogen exchanger 6 | Ps GGGTCTGTGTGCATGGGACT  Pa AGCAGCGTCACAATCCCTGA |
| DsaChr060031 | ATPase 1, plasma membrane-type | Ps TATGGAGGTGCTGGCTTCCG  Pa TCCCATTTGGCACTCAGGCA |
| DsaChr080167 | Plasma membrane ATPase | Ps CTGGGCTTGGCTATGGCAGA  Pa GCAGTGCTCGATGGTCTCCT |
| DsaChr150113 | V-type proton ATPase 16 kDa proteolipid subunit | Ps ATGGGTTTGCACACTTGGCG  Pa TCTCCCACGATTCCGATGGC |
| DsaChr110579 | Pyrophosphate-energized vacuolar membrane proton pump | Ps CGCTATGCTGCCCTACTGGT  Pa GAGGCGTTGGTGCTAATGGC |
| DsaChr090037 | Glucose-6-phosphate 1-dehydrogenase 1, chloroplastic | Ps TTCTATGAGGGCCTGCTGCC  Pa TGCCGATGAGGTCTCGGAAC |
| DsaChr040294 | Chlorophyll a-b binding protein of LHCII type I, chloroplastic | Ps TGGTTCAAGGCTGGTGCTCA  Pa GTGGCAATGATGCTCTGGGC |
| DsaChr050266 | Chlorophyll a-b binding protein CP29 | Ps CAACACTGAGAGGCCCGAGT  Pa ACGAAGTCGTTGGGCTTGGA |
| DsaChr010833 | Biotin carboxylase 1, chloroplastic | Ps GTGGAGGAGATCGGACTGCC  Pa GCCTGCTTCATGAGGGGGAT |
| DsaChr011057 | Acetyl-CoA carboxylase 1, cytosol | Ps TACCGGCAGCTCACCTATGC  Pa GTAGGAGGCAGCCAAGAGGG |
| DsaChr110486 | Glycerol-3-phosphate acyltransferase, chloroplastic | Ps CACCGATCCGATGTGCAAGC  Pa GCTGCATGGCAATGAGGGTC |
| DsaChr010659 | 1-acylglycerol-3-phosphate O-acyltransferase | Ps TATGTGGGCTTGGGAGCAGG  Pa GGCAGCCAGCTGATGGTAGA |
| DsaChr070135 | Stearoyl-[acyl-carrier-protein] 9-desaturase 2, chloroplastic | Ps GCTGCCTCAATCGCAAGACC  Pa TCTCGTCACGCACTCCATCC |
| DsaChr120549 | Phosphatidate phosphatase PAH1 | Ps TGTATCTGAGCAGCCGGTCG  Pa CGGCGCAGGATCATCTCTCT |
| DsaChr090216 | Diacylglycerol O-acyltransferase 2 | Ps GCTCCGTGGCTGTCATTGTG  Pa CACAACTCCATCCGCTCCCT |
| DsaChr120176 | diacylglycerol O-acyltransferase 1 | Ps GGCGCCTGGAGCTTGAAATC  Pa TACAGCCGCACTGGTGTTCA |
| DsaChr030703 | Diacylglycerol O-acyltransferase 2 | Ps CACGGCGTGTGTGATGTCAG  Pa TCCTCTTGGCCAACACCAGG |
| DsaChr010813 | Phospholipid:diacylglycerol acyltransferase | Ps AACTTGTTGAGGCGCTTGCC  Pa TGGTCAGCACCACCTTCTGG |
| DsaChr050146 | Phosphatidate cytidylyltransferase 4, chloroplastic | Ps GCCCTGGCATCCTTCATCCT  Pa AGCTAGGCAGCCATCCACAG |
| DsaChr120625 | Lysophospholipid acyltransferase 1 | Ps CTGGCAGAGGCAGGCTACAT  Pa ACCATACGGGTGCTCTCAGC |
| DsaChr040481 | Elongation factor 1-alpha  (Reference gene) | Ps CCTGCTGCATGCTCTGAACG  Pa TTGTAGGCGCCAGAGATGGG |
| Novelgene1135 | Transaldolase  (Reference gene) | Ps CTGCAGCCCGGACTTACTCA  Pa GCCACCAAGCCATCTGCATC |
